# Single-cell transcriptomics uncovers key immune drivers of vaccine efficacy in cattle

**DOI:** 10.1186/s12864-025-11915-0

**Published:** 2025-08-18

**Authors:** Annaleise Wilson, Pâmela A. Alexandre, Aaron M. Brice, Brad C. Hine, Aaron Ingham, Thibault P. R. A. Legrand, Caroline Royle, Dominic Niemeyer, Antonio Reverter, Stuart E. Denman, Ryan J. Farr

**Affiliations:** 1 CSIRO Health & Biosecurity, Australian Centre for Disease Preparedness, Geelong, VIC Australia; 2 CSIRO Agriculture & Food, Queensland Biosciences Precinct, St Lucia, QLD Australia; 3 CSIRO Australian Animal Health Laboratory, Australian Centre for Disease Preparedness, Geelong, VIC Australia; 4 CSIRO Agriculture & Food, FD McMaster Laboratory, Armidale, NSW Australia

**Keywords:** Adaptive immunity, Bovine immunology, Gamma-delta T cells, Innate immunity, Single cell sequencing, Transcriptome, Vaccine response

## Abstract

Comprehensive characterization of bovine immune cell populations is essential for improving animal welfare and disease resilience. We performed single-cell RNA sequencing on over 29,000 peripheral blood mononuclear cells (PBMCs) from Angus cattle stratified by delayed-type hypersensitivity (DTH), a proxy for the cellular immune response (Cell-IR). Unsupervised clustering identified major immune populations including CD4^⁺^ and CD8^⁺^ T cells, γδ T cells, B cells, monocytes, and dendritic cells. Differential gene expression suggests that low Cell-IR cattle have in elevated NKT inflammatory response, while high Cell-IR cattle have increased CD8- γδ T cell and pro-inflammatory myeloid activity. Intercellular communication analysis using CellChat highlighted pro-inflammatory cytokine cascades, particularly the IL-1β– IL-1R1 ligand-receptor interactions. This study provides a high-resolution atlas of Angus PBMCs and establishes a framework for linking immune cell composition with functional immune phenotypes in cattle.

**Supplementary Information:**

The online version contains supplementary material available at 10.1186/s12864-025-11915-0.

## Introduction

The health and productivity of livestock are essential for sustainable global agriculture. However, infectious diseases continue to pose a major challenge to cattle herds, resulting in significant economic losses through mortality, decreased performance, and the costs of treatment and prevention [1]. A comprehensive understanding of the bovine immune system, especially its cellular and molecular components, is critical for the development of effective disease control strategies [2].

Advances in single-cell RNA sequencing (scRNA-seq) have enabled high-resolution characterization of immune cell heterogeneity in production animals. This technology has been successfully applied to chickens [3] and pigs [4], and recent studies have applied it to cattle, advancing our understanding of bovine immunology [5–8]. For example, scRNA-seq of Holstein cattle peripheral blood mononuclear cells (PBMCs) identified cell-type-specific inflammatory responses to in vitro LPS stimulation, particularly NF-κB pathway activation in monocytes and dendritic cells [6]. Additionally, scRNA-seq analysis of bovine mesenteric lymph nodes identified a diverse range of dendritic and monocytic cell populations and provided insights into their distinct roles in antigen presentation and adaptive immune regulation [7].

Compared to other species, cattle possess unique immune characteristics which is likely an adaptation to their physiology and environmental exposures. For example, cattle have a high proportion of γδ T cells in peripheral blood in early life compared to humans, where αβ T cells dominate [9], suggesting that γδ T cells play a more prominent role in bovine immune responses, functioning as a major regulatory T cell subset [10]. Comprehensive characterization of PBMCs in meat-production breeds, such as Angus cattle, particularly under conditions relevant to natural infections or vaccination, has yet to be performed. Moreover, the role of cell-to-cell communication networks in shaping immune responses in bovine PBMCs has not been fully explored.

Vaccination remains a key tool in mitigating the risk of infectious diseases, yet the subsequent level and length of protection varies; a phenomenon that could reflect underlying differences in immune competence [11]. Animals with low immune competence not only exhibit poorer vaccine responses but also experience higher disease incidence and increased health-related costs, particularly during feedlot finishing in Angus cattle [12]. Identifying the genetic, cellular, and molecular determinants of immune competence holds promise for improving livestock health through selective breeding strategies that enhance disease resistance and vaccine efficacy. A phenotypic measure of immune competence in Australian Angus beef cattle has been developed, which is a combined metric of an animal’s ability to mount both cell-mediated (Cell-) and antibody (Ab)-mediated immune responses (IR) [13]. The Cell-IR and Ab-IR traits are moderately heritable; other factors such as differences in gene regulation, environment and prior pathogen exposure may be impacting the observed phenotypic variation [14].

In this study, we used single-cell RNA sequencing to profile bovine PBMCs and gain a deeper understanding of the peripheral immune system and its intercellular dynamics. This approach enabled us to map immune cell diversity and characterize both cell-type-specific gene expression and intercellular signalling. We focused on PBMCs collected four days after vaccination, a timepoint that captures early activation and differentiation within the innate and early adaptive immune compartments [15, 16], to explore molecular and cellular features associated with divergent cell-mediated immune responses (Cell-IR) in Angus cattle. To assess Cell-IR, we used the delayed-type hypersensitivity (DTH) response to secondary vaccination with a commercial multivalent clostridial and leptospiral vaccine (Ultravac^®^ 7in1, Zoetis). DTH is a well-established in vivo assay of cell-mediated immunity, reflecting antigen-specific T cell recruitment and activation at the site of antigen exposure. Higher DTH responses are generally indicative of a more robust immune system [17]. Single-cell transcriptomics is uniquely suited to dissecting the heterogeneity and dynamic nature of cell-mediated immune responses [18]. By comparing immune cell composition and gene expression profiles in animals with high versus low DTH responses, we sought to identify molecular markers of immune competence in cattle. These findings could inform the selection of disease-resistant animals and support breeding programs aimed at improving immune resilience in cattle.

## Materials and methods

### Animal cohort and immune phenotyping

The Angus steers (*n* = 67) used in this study were a subset of 5319 animals enrolled in the Angus Sire Benchmarking Program, as previously described [14]. All steers had received a primary vaccination with a commercial clostridial and leptospiral vaccine (Ultravac^®^ 7in1, Zoetis), administered subcutaneously (as per manufacturers recommendations) at marking (median age of 60 days old). The Ultravac^®^ 7in1 vaccine contained *Clostridium perfringens* type D (≥ 5 IU/mL), *Clostridium tetani* (≥ 2.5 IU/mL), *Clostridium novyi* type B (≥ 3.5 IU/mL), and *Clostridium septicum* (≥ 2.5 IU/mL) as ultrafiltered toxoids, as well as *Clostridium chauvoei* (0.3 mL/mL), *Leptospira borgpetersenii *serovar Hardjo type Hardjobovis (≥ 400 × 10^6^ organisms/mL), and *Leptospira interrogans* serovar Pomona (≥ 400 × 10^6^ organisms/mL) as formol cultures. The vaccine also contained an adjuvant consisting of aluminium salts and thiomersal as a preservative. All steers received a secondary subcutaneous vaccination of Ultravac^®^ 7in1 on the day of weaning. Steers were yard weaned with access to high quality hay and commercial weaner pellets for the duration of the testing period (Day 0–14). All procedures were minor, conducted on conscious animals, and did not require anaesthesia. No animals were euthanised for the purpose of this research.

To assess Cell-IR, baseline double skin-fold thickness (DSFT) of the caudal folds on each side of the tail were assessed with calipers at the proposed site of injection on Day 12 post vaccination. Immediately following baseline skin fold measurements, 100 µL of the Ultravac^®^ 7in1 vaccine was administered intradermally into the caudal fold on one side of the tail (test reaction) and 100 µL of a saline solution was administered intradermally into the caudal fold on the other side of the tail (control reaction). The magnitude of DTH reactions was assessed 48 h (Day 14) post injection using callipers. Each measure was taken three times, and the average values were used. Cell-IR was defined as the proportional increase in the test reaction compared to the control reaction on Day 14 after adjusting for the baseline (Day 12). Animals were ranked by their Cell-IR score and the four highest and four lowest ranking animals, that also had high sample viability (> 80% live cells) and concentration (> 1 × 10^6^ cells), were selected for scRNA-seq (*n* = 8). Differences in age and weight between high and low responder groups were assessed using an unpaired t-test.

### Single-cell isolation and sequenci

Whole blood was collected from the jugular vein using 8 ml lithium heparin tubes on Day 4 post vaccination with the Ultravac^®^ 7in1. The PBMCs were isolated using SepMate tubes (StemCell), following the manufacturer’s instructions. Cells were immediately frozen in Fetal Bovine Serum (FBS) with 10% DMSO at a cooling rate of 1°C/min. Samples were shipped on dry ice within two days of collection to the CSIRO Australian Centre for Disease Preparedness for long-term storage in liquid nitrogen. Prior to single cell barcoding, cells were pelleted by centrifugation at 300 g for 5 min and washed three times with 1X Phosphate Buffered Saline (PBS) containing 0.04% Bovine Serum Albumin (BSA; 400 µg/ml). Samples were barcoded using the 10x Genomics 3’ CellPlex Kit (v3.1) and pooled in equal concentration. scRNA-seq libraries were generated using the 10X Genomics Chromium Single Cell 3’ Reagent Kit v3. The final libraries containing the P5 and P7 primers were used in Illumina bridge amplification and were assessed for quality and DNA concentration using a High Sensitivity DNA chip on a BioAnalyzer 2100 (Agilent Technologies). Sequencing was performed using the Illumina NovaSeq 6000 platform, with 28 bp (read 1) and 90 bp (read 2) length reads.

### Quality control, dimensionality reduction, and cell clustering

Sequencing reads were analysed using the Cell Ranger v7.2.0 software. FASTQ files were aligned to the *Bos taurus* ARS-UCD1.2 genome with the Ensembl 98 annotation release using STAR, implemented in the Cell Ranger count pipeline, which also conducts the subsequent filtering and counting of cell barcodes and Unique Molecular Identifiers (UMIs). Reads generated by barcode-associated cells, which passed the pipeline-internal QC, were quantified and used for establishing a gene-barcode matrix. All downstream analyses were accomplished using the Seurat R package v5.0.1 [19].

Cells which contained less than 200 or greater than 3,000 gene features, and less than 7% mitochondrial-DNA derived gene content, were filtered from the dataset. Samples were integrated using the FindIntegrationAnchors and IntegrateData functions. Samples were normalized and scaled prior to course unsupervised clustering using a resolution of 1.5. Clustering was done with the FindNeighbors and FindClusters functions using the first 50 PCs from the dimensionality reduction step. Visualization of the cells was performed using the UMAP algorithm as implemented by the Seurat RunUMAP function. Cell types were manually curated using canonical marker genes identified using the FindConservedMarkers function. Population level analyses were performed by sub-setting clusters by population type (i.e. T cells) and reperforming the “FindClusters” function at higher resolution (> 1.5).

### Cell communication analysis

Cell-cell communication analysis was performed using the CellChat v2 package [20] to infer ligand-receptor interactions, calculate communication probability, and identify significant signalling pathways between major cell types in the peripheral blood. As there is no bovine database in CellChat package, the human database was used. The analysis was conducted on all eight samples.

### Analysis of differential cell population and gene expression

Differences in cluster proportions between high and low responders were assessed using scProportionTest (https://github.com/rpolicastro/scProportionTest/releases/tag/v1.0.0), with significance defined as an absolute Log_2_ Fold Enrichment > 0.58 and a false discovery rate (FDR)-adjusted *P* < 0.05. Permutation plots were generated using the ggplot2 package [21]. The DEseq2 package [22] was used to detect differentially expressed genes (DEGs) for each cluster in each pairwise comparison between high and low responders. Differences were considered significant with an FDR-adjusted *P* < 0.05. Gene set enrichment analysis was performed using the WebGesalt tool [23] and the biological process geneontology functional database, with an FDR-adjusted *P* < 0.05 and the weight set cover redundancy removal parameter.

## Results

### Single cell profiling of peripheral blood mononuclear cells from Angus steers with divergent cell-mediated immune responses to vaccination

**Fig. 1:**
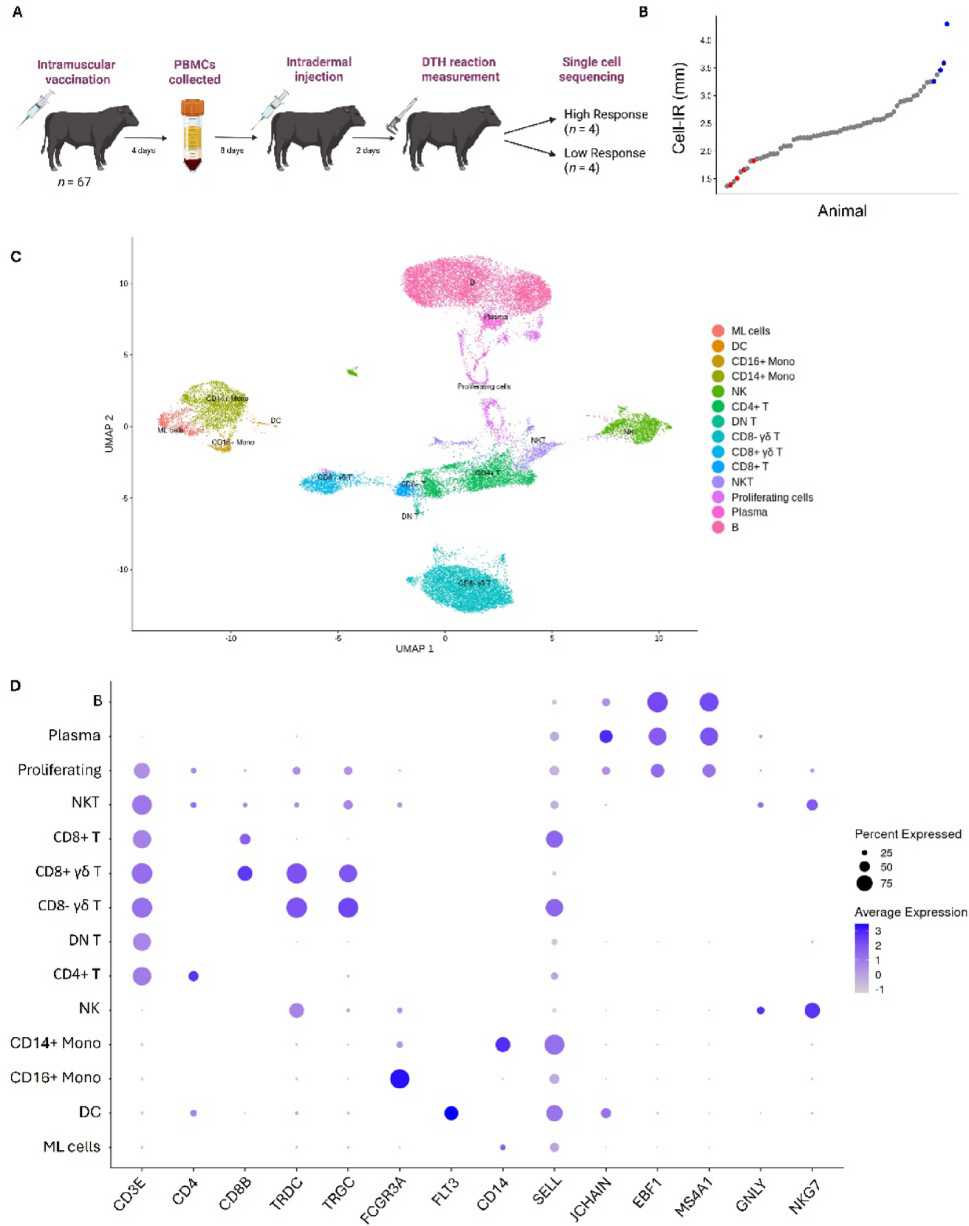
Study design and cluster analysis of bovine PBMC using single-cell sequencing (scRNA-seq). **A** Schematic representation of the study design. Peripheral blood mononuclear cells (PBMCs) were collected from Angus steers (*n* = 67) that were delayed-type hypersensitivity tested against a multivalent clostridial and leptospiral vaccine. **B** The distribution of cell-mediated immune responses (Cell-IR) scores across individual steers (dots). Steers classified as high and low Cell-IR which were selected for scRNA-seq are highlighted in blue and red, respectively. **C** Uniform Manifold Approximation and Projection (UMAP) of individual cells indicated the clustering of PBMCs into 14 distinct cell types. Each point represents a single cell, colour-coded by cell cluster identity, with course immune cell type annotation. **D** Dot plot depicting the expression of canonical marker genes for each identified cluster. The size of the dots corresponds to the proportion of cells expressing the gene in each cluster, while the colour intensity reflects the mean normalised gene expression

We performed scRNA-seq to characterise bovine PBMC heterogeneity and assess differences in gene regulation between Angus steers with divergent immune responses. In total, 67 steers were assessed for Cell-IR on Day 14 of weaning (Fig. 1A). Animals were ranked by their Cell-IR score, and PBMCs that were collected at four days post-vaccination from the four highest (blue) and four lowest (red) scoring animals were used for scRNA-seq (Fig. 1B). Animals selected for sequencing had Cell-IR phenotypes within top or bottom 12% of Angus steers (*n* = 5319) enrolled in the Angus Sire Benchmarking Program. There were no statistical differences (*P* > 0.05) between high and low response groups for age (median 222 days and 223 days, respectively) or weight (median 292 kg and 318 kg, respectively) at the time of the experimentation.

In total, 32,447 single cells from eight samples were sequenced, producing an average of 19,282 reads per cell (Supplementary Table 1). After quality filtering, removal of cells with uncertain cell type annotation, we obtained transcriptomes for 29,771 single cells and annotated 17,075 genes across the dataset. Immune cell populations were identified based on their expression of marker gene expressions following normalisation and clustering (Fig. 1C). Fourteen coarsely defined cell populations, including B lymphocytes (30.67%), CD4+ T (12.67%), CD8+ T (1.93%), CD8- γδ T (21.94%), CD8+ γδ T (4.57%), Natural Killer T (NKT; 3.85%), Natural Killer (NK; 7.09%), plasma B cells (0.44%), CD14+ monocytes (8.13%), CD16+ monocytes (0.89%), double-negative T (DN T; 0.41%), dendritic cells (DCs; 0.22%), myeloid-like cells (ML cells; 2.62%) and proliferating cells (4.57%), which were classified according to canonical markers identified using the FindMarkers function in Seurat (Fig. 1D). Proliferating cells as indicated by their high expression of genes involved in cell replication including MKI67, SMC2 and MCM7.

### T cell cluster analysis

**Fig. 2:**
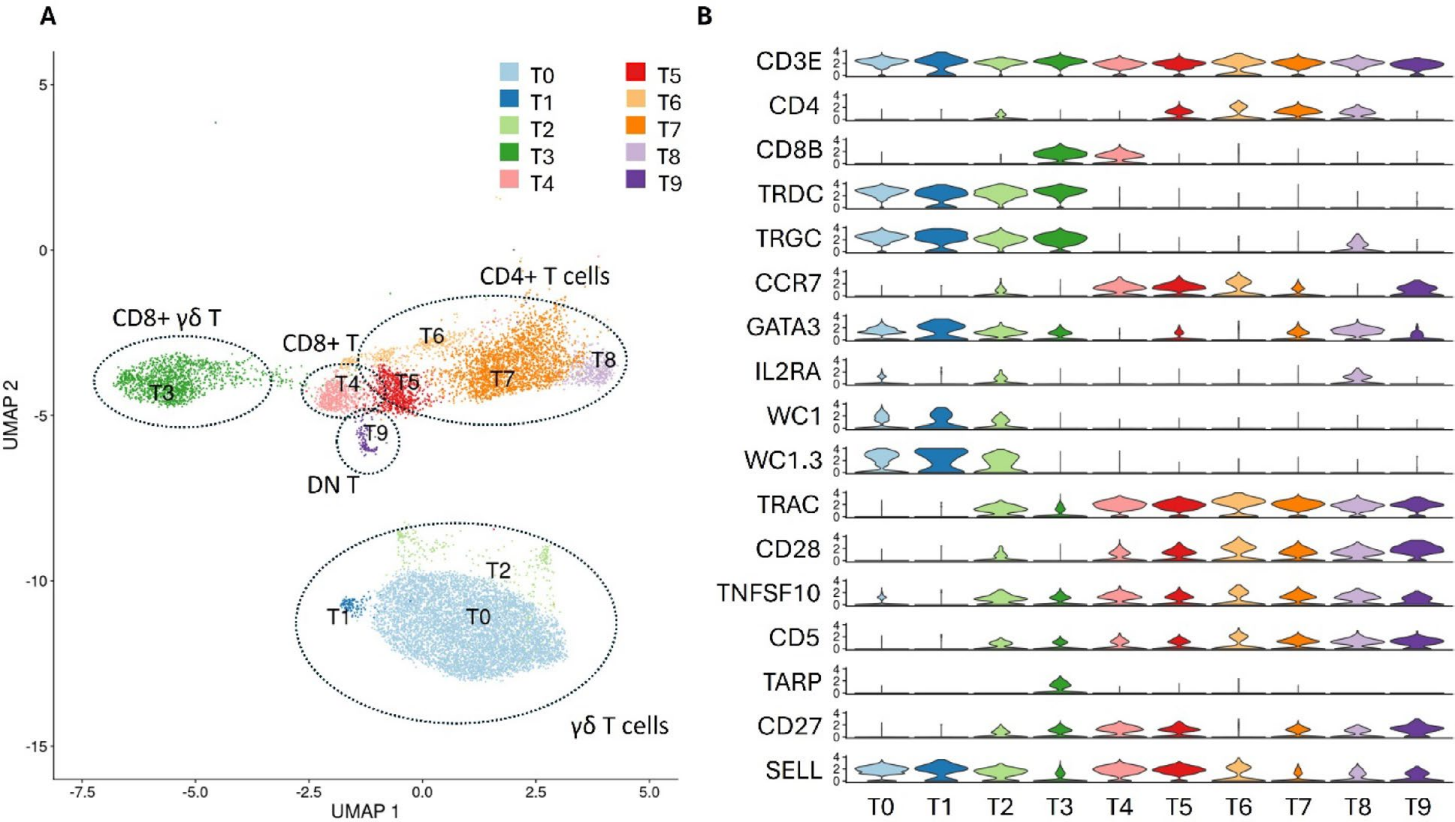
T cell populations. **A** UMAP showing T cells subtypes based on their gene expression profiles. **B** Violin plot of genes differentially abundant across one or more T cell population

Ten putative T-cells sub-populations were identified based on their CD3E expression (Fig. 2A). Among these, were four distinct clusters of γδ T cells (T0-T3), which expressed the T-cell receptor δ (TRDC) and γ constant (TRGC) genes. Notably, a subset of γδ T cells (T3), representing 17.36% of this subtype, also expressed CD8B and TCR γ Alternate Reading Frame Protein (TARP), indicating that they are CD8+ γδ T cells [24]. The proximity of this cluster to CD8+ T cells (T4) suggests that this cluster has a distinct molecular and, potentially, functional profile to CD8- γδ T cells (T0-T2). A key molecular distinction between the two γδ T cell subsets is the upregulation of Zinc finger protein Helios (IKZF2) in CD8+ γδ T cells. In comparison, CD8- γδ T cells uniquely expressed transmembrane glycoproteins Workshop Cluster (WC) 1.1 and WC1.3. There were four CD4+ T cell clusters (T5–T8), although only a portion of cells in T6 expressed CD4+. Cluster T5 was characterized by high expression of CCR7, CD27, SELL (CD62L), and LEF1, consistent with naive CD4+ T cells [25, 26]. Despite their predicted naive phenotype, T5 cells also demonstrated upregulation of metabolic activation pathways, with gene ontology analysis of the top 100 genes over-expressed in this cluster saw enrichment for ribosomal small subunit assembly (GO:0000028), large subunit assembly (GO:0042273), and cytoplasmic translation (GO:0002181). This suggests that these cells may be transitioning to an activated or proliferative state post-vaccination, although activation marker IL2RA (CD25) expression remained low four days post-vaccination. Cluster T6 displayed low expression of CD27 and moderate CD62L and CCR7, suggesting these possess a T central memory cell phenotype. In contrast, T7 and T8, characterized by high CD27 and GATA3 expression but low CCR7 and CD62L levels, appear to represent Th2 effector cells. Small subset of T cells (T9) lacked both CD4 and CD8 expression, a phenotype associated with DN T cells.

Interestingly, the proportion of CD8- γδ T cells was significantly elevated in high Cell-IR phenotype animals (0.70 Log_2_ Fold Enrichment), whereas CD8+ γδ T proportions were similar (Supplementary Table 2). 499 genes were differentially expressed (DEGs) in CD8- γδ T cells between high and low responders, compared to only 38 DEGs between CD8+ γδ T cell populations (Supplementary Dataset 1). Enriched pathways between CD8- γδ T cell populations included those for signalling (GO:0023052), response to stimulus (GO:0050896), cell population proliferation (GO:0008283), and hemopoiesis (GO:0030097), highlighting their potential involvement in vaccine-induced immune responses. Within the broad pathways of ‘signalling’ and ‘response to stimuli’, were genes involved in ‘positive regulation of T-helper 1 (Th1) cell differentiation’, namely RIPK2 (-0.40 Log_2_FC) and ANXA1 (0.55 Log_2_FC), as well as ‘nucleotide-binding oligomerisation domain containing 1 (NOD1) signalling regulation’, denoted by genes RIPK2 and NFKBIA (-0.47 Log_2_FC). In addition to NFKBIA, IkB proteins NFKBIB (-0.37 Log_2_FC) and NFKBID (Log_2_FC -0.88) were enriched in high responders, as well as NF-kB (NFKB1; -0.27 Log_2_FC). No significant differences in the relative proportions of CD4+, CD8+, or DN T cells were observed between high and low Cell-IR phenotype animals. Additionally, pathway enrichment analysis revealed no major differences in gene expression between conditions. Interestingly, ENSBTAG00000052397, an IL-32 link protein, was up-regulated in low responders in CD4+ T (5.24 Log_2_FC), CD8+ T (3.23 Log_2_FC), DN T (6.70 Log_2_FC), CD8+ γδ T (3.30 Log_2_FC) and CD8- γδ T (4.62 Log_2_FC).

### Myeloid pro-inflammatory cytokine expression is associated with high cell-mediated immune response to vaccination

**Fig. 3:**
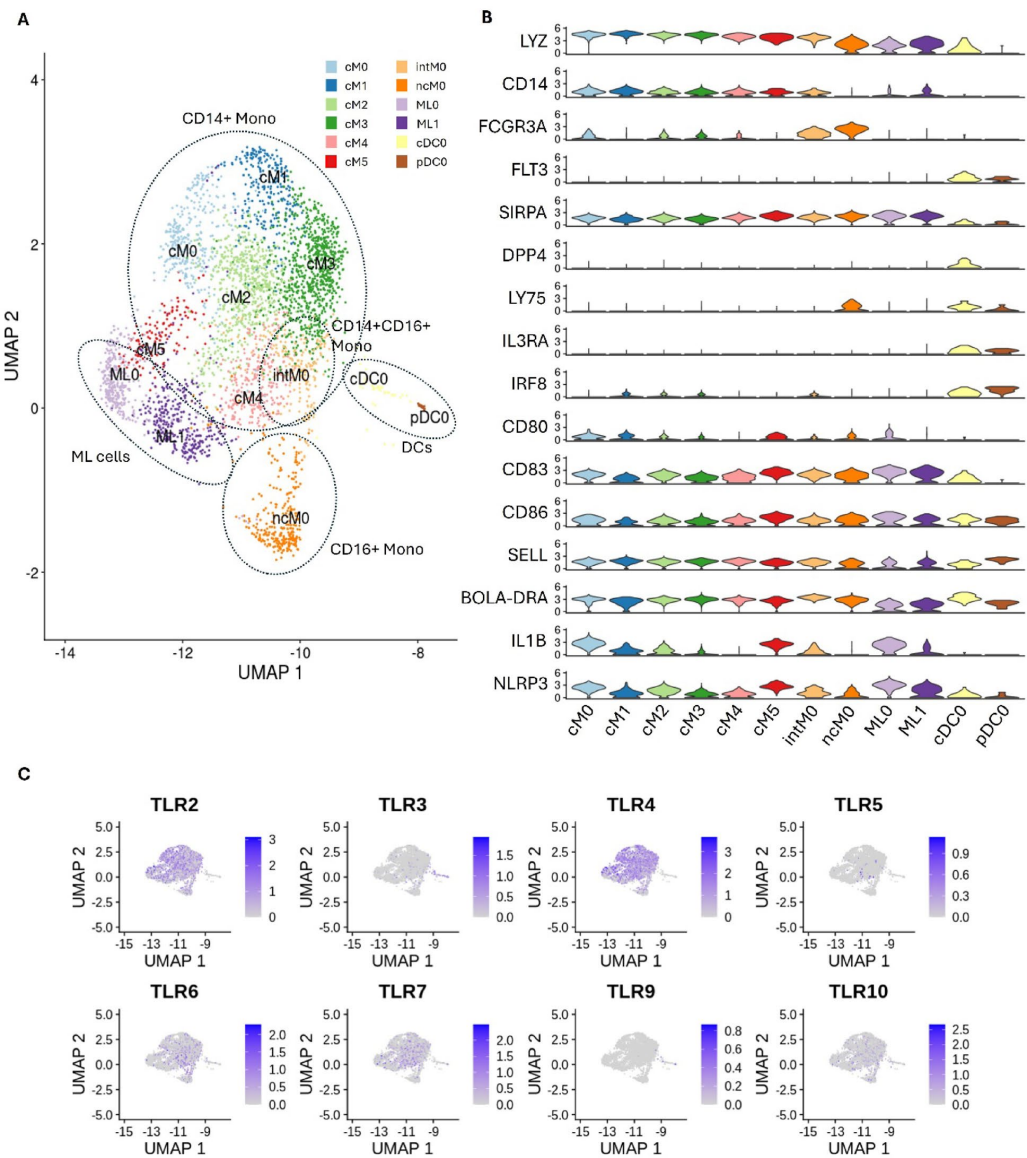
Myeloid cell populations. **A** UMAP of monocytes, myeloid-like cells and dendritic cells (DCs) clustered based on their gene expression profiles. **B** Violin plot of genes differentially abundant across one or more cell populations. **C** UMAP demonstrating Toll-Like Receptor (TLR) expression across myeloid cell populations. Colour scale represents log-normalized expression level of the indicated gene in individual cells

There was extensive heterogeneity amongst bovine monocyte populations, not only in their expression of CD14 and CD16 but also in their activation status and functional profiles (Fig. 3A). Monocyte clusters were found to be broadly categorized by their expression of cell surface markers; CD14^hi^CD16^lo^ classical (cM0– cM5), CD14^hi^CD16^hi^ intermediate (intM0) and CD14^lo^CD16^hi^ non-classical (ncM0). Additionally, difference in transcription factors and activation markers further defined these clusters (Fig. 3B). Clusters cM0 and cM5 exhibited an expression profile predictive of highly pro-inflammatory phenotype. However, cM5 demonstrated distinct functional specialization with higher expression of CCL16, CD80, and the APC activation marker CD83, suggesting enhanced pro-inflammatory and antigen-presenting roles compared to cM0. Cluster cM1 possessed a unique cell expression profile compared to other monocyte clusters. Gene ontology analysis of its top 100 overexpressed genes revealed pathways linked to neutrophil aggregation (GO:070488), neutrophil-mediated killing (GO:0070945), neutrophil chemotaxis (GO:0030593) and innate immune response (GO:0045087). High expression of S100 proteins and defensins (DEFB1, DEFB4A, DEFB7, DEFB10 and DEFB13) suggests that these monocytes are specialized for antimicrobial defence and may represent cells in early macrophage differentiation. Unlike M5, ncM0 did not express IL-1β but exhibited robust expression of CCL16, suggesting a regulatory role in immune modulation, pathogen clustering, and cell recruitment rather than initiating pro-inflammatory responses.

We also explored the distribution of Toll-like receptors (TLR) expression, which was varied across cell types (Fig. 3C). As expected TLR2 and TLR4, co-receptors for CD14, were significantly expressed across CD14 + monocyte populations. Both DC populations express high TLR3, with low expression of TLR6 and TLR7. Expression of TLR9 was minimal across DC populations. Both TLR6 and TLR7 are enriched in inactivated monocytes clusters, while expression was considerably lower in cluster M0 and both myeloid-like clusters. Interestingly, while TLR10 was predominately expressed in intM0, it was sporadically detected across all other cell types. Neither TLR1 nor TLR8 was detected in any PBMC population, aligning with their previously reported restricted expression patterns in cattle [27].

Two dendritic cell clusters were identified: DC0 and DC1 (Fig. 3A). Both expressed FLT3, confirming their dendritic cell identity. DC0 uniquely expressed DPP4, suggesting a conventional dendritic cell (cDC) phenotype, while DC1 expressed higher levels of IL3RA and CD4 (CD123), alongside lower BOLA-DR expression compared to DC0, consistent with a plasmacytoid dendritic cell (pDC) phenotype [27].

Lastly, two clusters observed were classified as myeloid-like cells (ML0 and ML1), due to their proximity to monocyte clusters and expression of LYZ, SIRPA (CD62L), CD86, and MHC Class II. However, this cell type lacked definitive markers for monocytes (CD14, CD16) or dendritic cells (FLT3, IRF8) which prevented a definitive annotation. Both ML0 and ML1 were characterized by high expression of CD83 and IL-1β, indicative of an activated and pro-inflammatory state. Although constitutively expressed across both clusters, MHC Class II expression was slightly lower compared to monocyte and DC clusters.

**Fig. 4:**
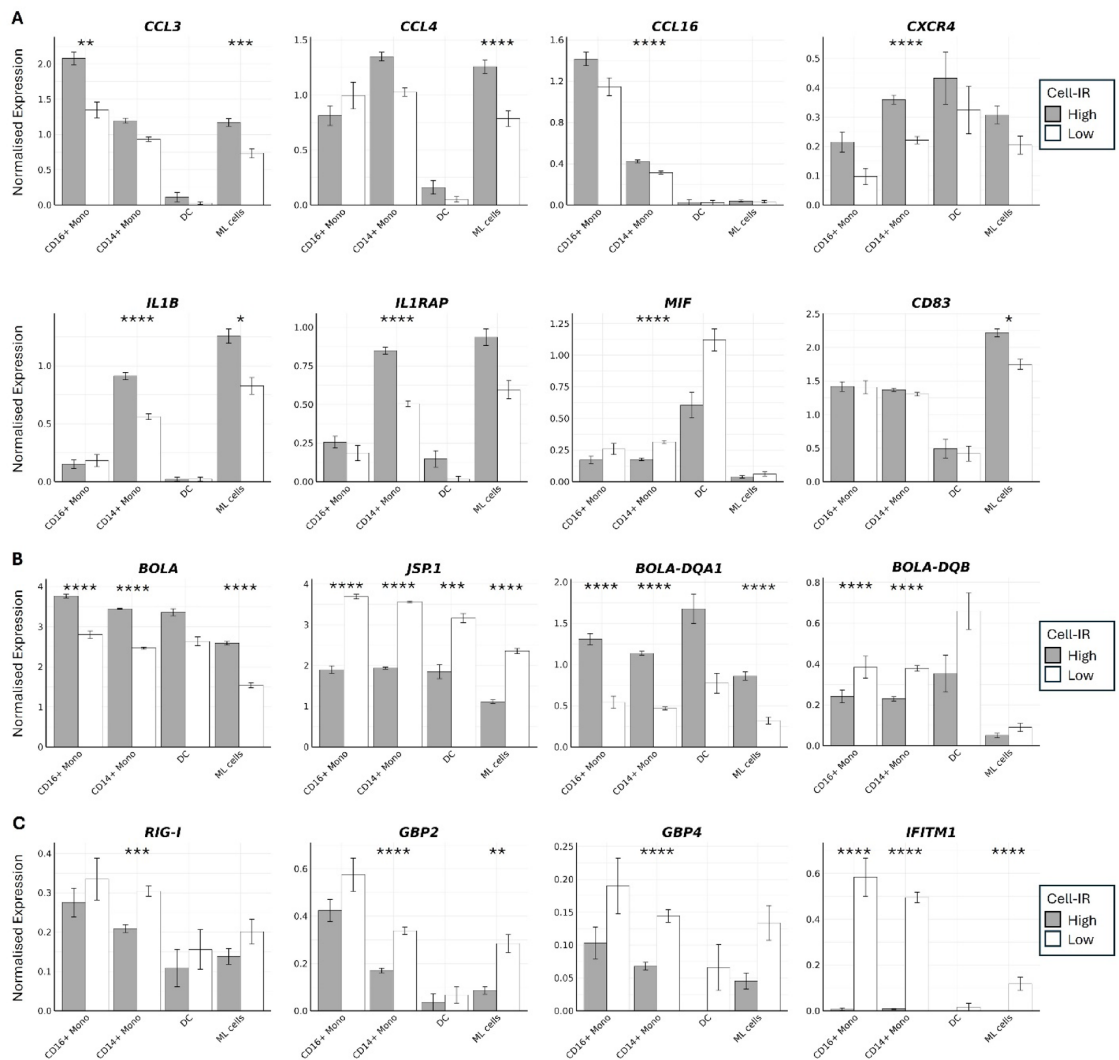
Differences in transcription of **A** cytokine signalling, chemokine signalling and immune activation, **B** major histocompatibility complex II and **C** interferon-inducible genes between animals with high and low Cell-IR per myeloid cell cluster identified using DESeq2. * *q* < 0.05, ** *q* < 0.005, *** *q* < 0.0005, **** *q* < 0.00005

A detailed analysis of myeloid cell composition and transcriptional profiles between animals with high and low Cell-IR immune responses to vaccination revealed significant differences in both cellular abundance and gene expression. CD16^⁺^ monocytes (1.0 Log_2_ Fold Enrichment) and myeloid-like cells (1.1 Log_2_ Fold Enrichment) were markedly enriched in high responders, while CD14^⁺^ monocytes were moderately enriched (0.38 Log_2_ Fold Enrichment; Supplementary Table 2). The CD14^⁺^ monocyte populations saw significant transcriptional changes, with 212 genes upregulated and 242 genes downregulated in high responders. Notably, genes encoding chemokines and their receptors, including CCL4 (MIP-1β), CCL16 (HCC-4), CXCR4, IL-1β, IL-1RAP and CLEC4A were upregulated in high responders, although expression of CLEC4A was minimal (Fig. 4A). Chemokine and cytokine expression was also elevated in ML cell populations of high responders (Fig. 3A), including IL-1β and CCL3, along with activation marker CD83. Interestingly, macrophage migration inhibitory factor (MIF) was upregulated in the CD14^⁺^ monocytes of low responders (Fig. 4A). Only one gene, JSP.1, was differentially expressed between DC populations of high and low responders (Fig. 4B), likely due to the small cell numbers sequenced in this dataset. Considerable variation was also observed in the expression of MHC Class I genes across all cell types. For instance, BOLA (BOLA-2*070:01)) was upregulated in high responders, whereas JSP.1 (BOLA-3*00201) was preferentially expressed in low responders. Among professional antigen-presenting cells (APCs), MHC Class II genes, including BLA-DQA1, BLA-DQA2, and BLA-DQB, were differentially expressed, with higher levels observed in high responders (Fig. 4B). Lastly, a small number of interferon-inducible genes including RIG-I, GBP2, GBP4 and IFITM1 were up-regulated in low responders, particularly in CD14+ monocytes, although expression levels of these genes were low across all cell types (Fig. 4C).

### Partitioning of B lymphocyte populations is related to expression of transcriptional regulators Sox5 and Bach2

**Fig. 5:**
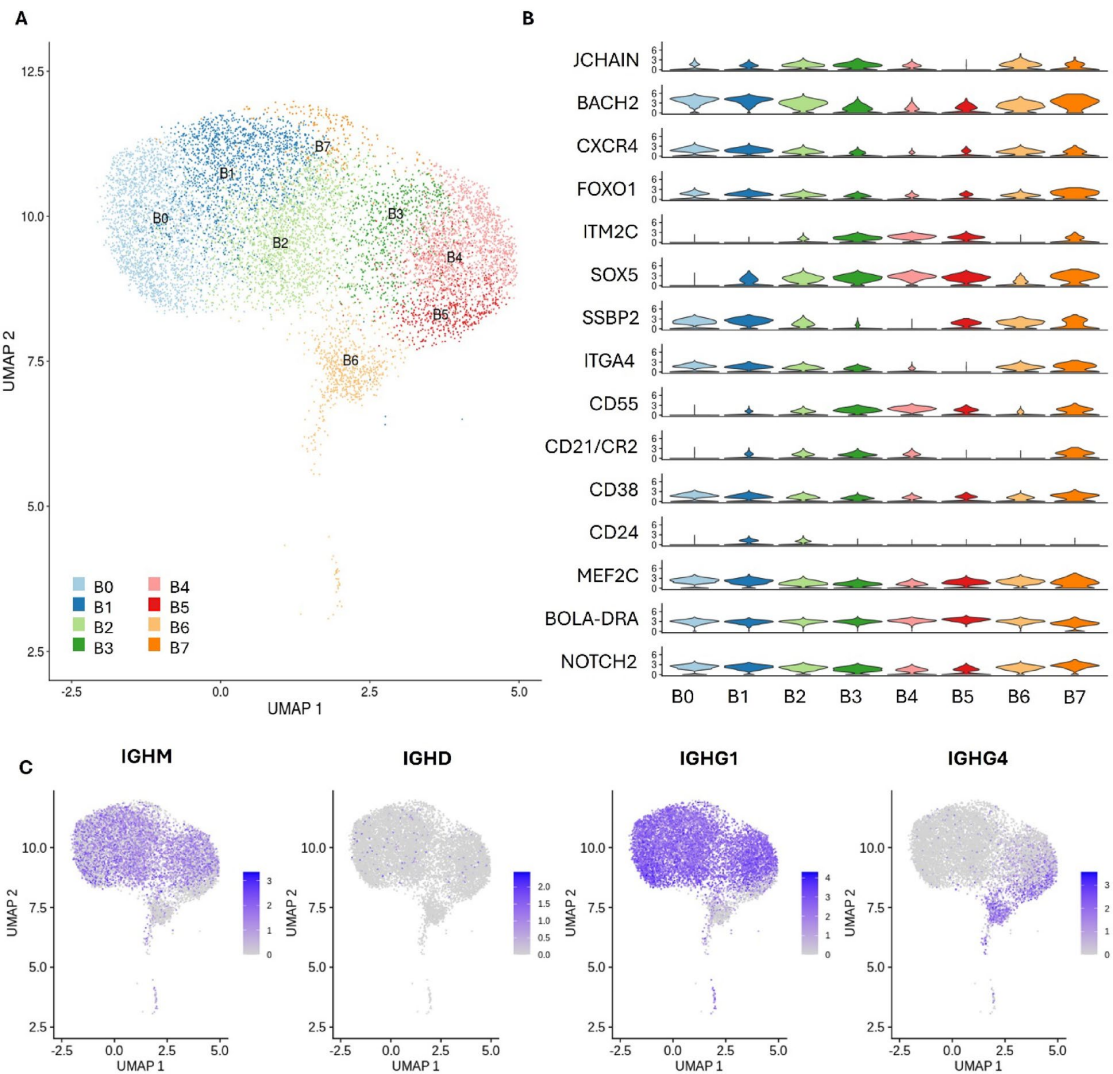
B lymphocyte and plasma cell analysis. **A** UMAP of B lymphocytes and plasma cells clustered based on their gene expression profiles. **B** Violin plot of genes differentially abundant across cell populations. **C** Expression of immunoglobulin genes across B cells. Colour scale represents log-normalized expression level of the indicated gene in individual cells

Eight B and plasma cell clusters were identified, although separation of these clusters based on their expression profile was less distinct than other cell populations. This was mainly driven by expression of regulatory genes and transcription factors that influence B cell differentiation (Fig. 5A, B). Among these, SOX5 and BACH2 emerged as key markers for distinguishing different subsets. Transcriptional repressor BACH2, known to be highly expressed in naïve B cells, moderately expressed in memory B cells, and downregulated in plasma cells [28], was found to be enriched in B0 and B1 clusters. Both clusters were CD21^low^, consistent with a memory B cell subset [29], however the high expression of BACH2 and SSBP2, the latter of which is involved in stem cell maintenance [30], and low expression of JCHAIN compared to other subsets suggests that these cluster are at an early stage of development. BACH2 expression appeared to be inversely related to SOX5 expression. Clusters B2 and B3 both expressed JCHAIN and SOX5, highly expressed in plasmablasts [31]. A distinguishing feature of clusters B3, in addition to B4 and B5, was the expression of ITM2C, a gene selectively expressed in antibody-secreting cells in mice and humans [32]. In contrast, B2 cells, which expressed both SOX5 and BACH2, suggests that these cells may be at an earlier stage of activation and differentiation.

All clusters express an Immunoglobulin Heavy Chain Gamma isoform (Fig. 5C), either IGHG1 (ENSBTAG00000050723; Clusters B0– B4, B7) or IGHG3 (ENSBTAG00000055240; Clusters B5–B6). The gene encoding for IGHM (ENSBTAG00000001219), was sporadically expressed across clusters B0 to B5, suggesting that IgM+ B memory cells in this dataset may have similar gene expression profiles to isotype-switched B cells. Interestingly, CD38 was low in IgG4+, JCHAIN + cells.

As there was significant overlap in the expression profiles of B cells clusters, differences in gene expression between animals high and low DTH response were performed at three levels: B memory cells (B0, B1, B4, B5), plasmablasts (B2, B3) and plasma cells (B6). When comparing the gene expression of B memory cells 557 genes were found to be DE, 174 up-regulated and 383 down-regulated in high responders. Despite this, there were no enriched gene ontology pathways. Between plasmablast populations there was 261 genes DE, 164 upregulated and 97 downregulated in high responders. As with B memory cells, no enriched pathways were identified. Lastly, between plasma cell populations there were 615 genes DE with 269 up regulated and 346 downregulated in high responders. Enriched pathways included “GO:0050764 Regulation of phagocytosis”, “GO:0002181 Cytoplasmic translation”, “Cellular response to chemical stimulation” and “GO:0009605 Response to external stimulus”.

### NK and NKT cells population heterogeneity is driven by cytotoxic effector gene expression

**Fig. 6:**
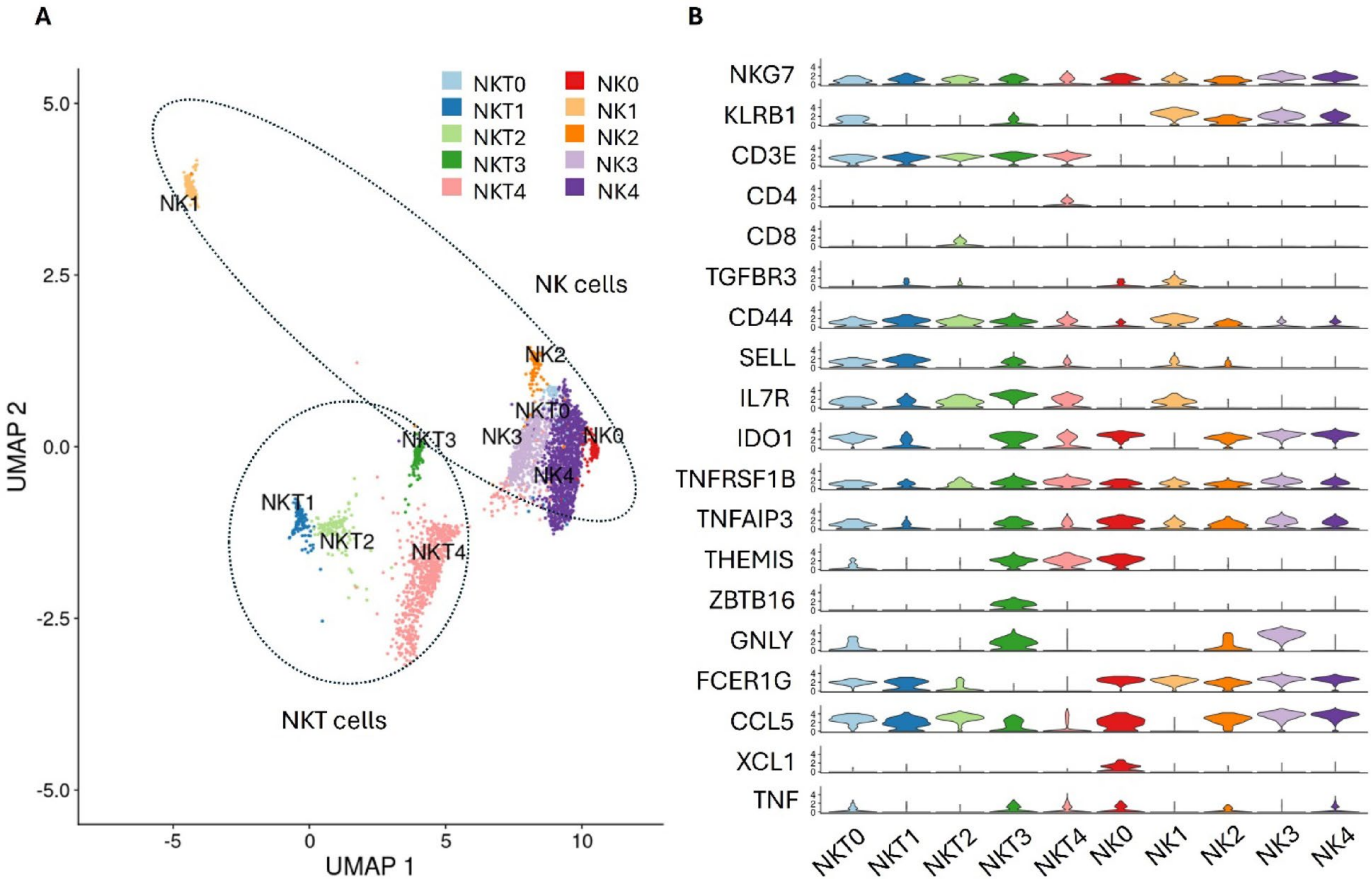
NK and NKT single cell analysis. **A **UMAP of NK and NKT cells clustered based on their gene expression profiles. **B** Violin plot of genes differentially abundant in one or more cell population

Five distinct NKT cell subtypes, designated NKT0, NKT1, NKT2, NKT3, and NKT4, were identified based on their unique transcriptional profiles (Fig. 6A). All subtypes uniformly expressed the cytotoxic markers NKG7 and CD3E, confirming their classification as NKT cells (Fig. 6B). NKT subtypes exhibited differential expression of the co-receptors CD4 and CD8, with NKT4 exclusively expressed CD4, NKT2 expressed CD8, all other subtypes were CD4-CD8-. No NKT subtype expressed both co-receptors, indicating a potential functional divergence between these subsets. Consistent with previous reports [33], CD56 was not found to be expressed in any NK or NKT subsets in bovine blood. All NKT subtypes expressed TNFRSF1B, a receptor involved in TNF-mediated signalling, and all NKT clusters except NKT2 expressed TNFAIP3. Cluster NKT3 likely represents Type I NKT or invariant NKT (iNKT) cells owing to the expression ZBTB16 (PLZF) and T cell receptor V gene (ENSBTAG00000049803), which is not expressed in other NKT or other traditional T cells subsets [34]. As with NKT cells, there were five subtypes of NK cells (NK0– NK4), that varied based on their expression of cytotoxic and pro-inflammatory cytokine expression (Fig. 6A). NK0 express high levels of TNF and CCL5 and uniquely expressed XCL1. On the other hand, NK1 cells lack expression of the aforementioned cytokines, and has a profile suggestive of a suppressive function, including TGF-β signalling.

NKT cells are the only cell type across all PBMC populations that were at greater proportions in low Cell-IR animals (1.07 Log_2_ Fold Enrichment). In addition, a total of 136 genes were DE, including 65 up-regulated and 72 down-regulated in high responder animals. Among enriched pathways, include “GO:0071674 mononuclear cell migration”, “GO:0001775 cell activation” and “GO:0030154 cell differentiation”. Opposed to findings for monocyte populations, CCL3 (1.51 Log_2_FC) was up-regulated in low responders. Genes highly expressed in low responders also suggest that they are potentially Th1 polarised, with top upregulated genes including Interferon-induced transmembrane protein 3 (IFITM3; 5.9 Log_2_FC), JSP.1 (2.16 Log_2_FC) and Annexin A1 (ANXA1; 1.32 Log_2_FC). Conversely, NKT cells from high responders upregulated genes that appear to be involved in immune suppression *VA2W* (-5.89 Log_2_FC), *PAPPA* (-2.87 Log_2_FC) and CPT1C (-2.56 Log_2_FC).

### Cell-to-cell signalling is predicted to be largely driven by myeloid cell clusters

**Fig. 7:**
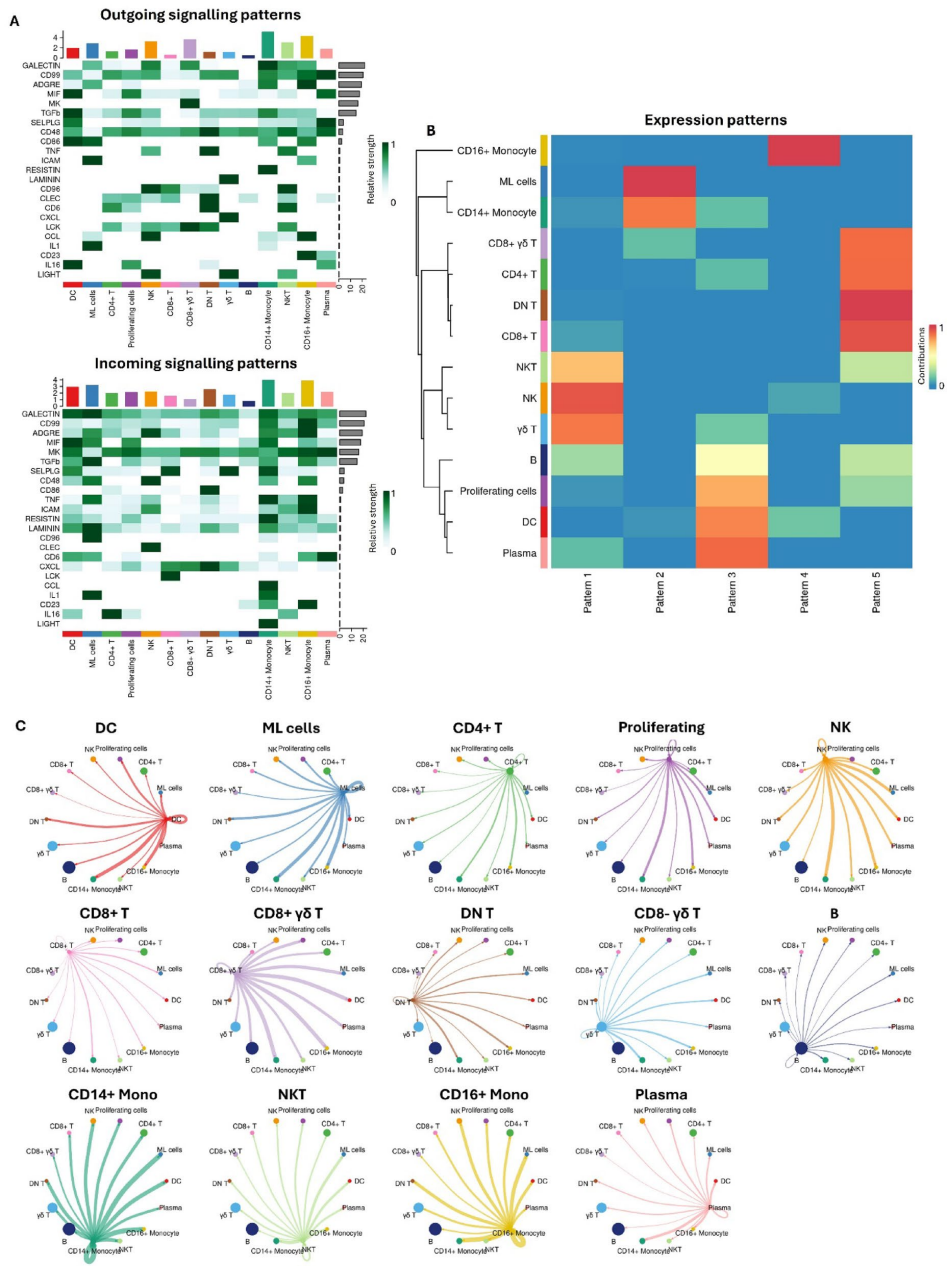
Cell population communication analysis. **A** Heatmap depicting significant outgoing and incoming ligand-receptor interactions between PBMC cell populations. The colour intensity represents the overall signalling strength computed by CellChat based on the expression of ligand-receptor pairs and the number of significant interactions. **B** Clusters based on communication patters between cell types. The colour intensity represents the contribution of each pathway to the overall signalling network. **C** Outgoing communication strength and receiver patterns for the 14 cell types in bovine PBMCs

We applied CellChat to predict significant cell-cell communication pathways at four days post vaccination by identifying overexpressed ligand-receptor pairs among various cell clusters. This analysis revealed 47 significant ligand-receptor pairs across the 14 cell populations examined, which included signalling pathways like TGFβ, TNF, CXCL, CCL, and MIF, as well as cell-cell communication pathways involving galectin, CD99, CD6, and CD48 (Fig. 7A). The strongest outgoing signalling patterns were observed in CD14+ monocytes, CD16+ monocytes, and CD8+ γδ T cells. Incoming signalling patterns were most prominent in CD14+ and CD16+ monocytes, ML cells and DC. Inference of expression communication patterns suggested that ML cells shared similar pattern to CD14+ monocytes despite not expressing CD14 (Fig. 7B). Cells expressing CD16+ clustered separately to other myeloid populations and DC cells were more closely related to plasma cells, B cells, and proliferating cells. Interestingly, CD8+ γδ T cells share more similar profiles to αβ T cells populations where as CD8- γδ T cells cluster with NK and NKT cells. Outgoing communication by CD8+ γδ T are primarily received by myeloid cells populations, B cells and plasma cells (Fig. 7C). Significant interactions were observed between CD14+ monocytes, CD16+ monocytes and ML cells and all other cell types but also performed significant “self-talk”.

Our findings also highlighted that significant outgoing TNF signalling patterns were largely driven by DN T cells, NK cells, and NKT cells (Supplemental Fig. 1), with the main receiving populations being CD14+ and CD16+ monocytes and ML cells, and to a lesser degree, CD4+ T and both γδ T populations. NK cells emerged as the primary targets of C-type lectin receptor (CLEC) signalling, while the CXCL signalling pathway indicated extensive crosstalk between γδ T cells and other cell types, predominantly with CD8+ γδ T, CD8+ T, and DN T cells. A particularly significant ligand-receptor pair identified was CXCL12-CXCR4, a crucial driver of these interactions. Notably, CD8+ γδ T cells did not contribute to CXCL-mediated outgoing signals. Instead, CD8+ γδ T cells were unique in their expression of midkine (MK), a ligand linked to cell proliferation, survival, tissue repair, and angiogenesis, as well as anti-apoptotic activities [35]. In contrast, the expression of CXCL signals by CD8^⁻^ γδ T cells, present in a higher proportion in high Cell-IR phenotype animals, suggests a heightened role in chemotactic functions, potentially orchestrating immune cell recruitment and organization at sites of inflammation or infection. As previously mentioned, IL-1 signalling is primarily performed by CD14+ monocytes and ML cells, consistent with their role in initiating inflammatory responses. The key targets of IL-1 secretion are also CD14+ monocytes and ML cells, indicating that IL-1 axis is central to monocyte and dendritic cell communication, rather than the broader cell-to-cell interactions typically seen in generalized inflammatory responses. IL-16 signalling is primarily performed by pDCs, plasma cells and DN T cells with CD4+ T, NK and DCs as receivers. DCs, along with plasma cells, are also a predicted to secrete MIF which along with MK, is an abundantly secreted immunosuppressive signals, which is predicted to interact with CD74 and ACKR3 receptors on other monocytes and ML cells.

## Discussion

Characterizing bovine PBMC populations is challenging due to the high degree of cellular heterogeneity. Traditional approaches, such as flow cytometry, are commonly used for identifying immune cell subsets; however, they depend on the availability of species-specific antibodies, which are less well-characterized in cattle compared to humans and mice. To overcome these limitations, this study employed scRNA-seq to comprehensively profile bovine PBMCs, providing a high-resolution map of cell states and transcriptional programs. These findings offer a valuable resource for future research focused on identifying biomarkers, refining selective breeding strategies, and developing targeted vaccines or therapeutics to enhance disease resistance in cattle.

We identified immune cell populations, subsets and transcriptional profiles in bovine PBMCs not currently described in other studies. For instance, we characterised a myeloid-like cluster that expressed markers common to mononuclear phagocytes (monocytes, macrophages and dendritic cells), LYZ, SIRPA, CD86, and MHC Class II. Although, expression of SIRPA and CD86, as well as CD83, appeared to be enhanced in these clusters. CD86, along with CD80, are co-stimulatory molecules that enable monocytes to activate lymphocytes [36], CD83 also aids in lymphocyte regulation [37], while SIRPA inhibits phagocytosis by binding to the CD47 expressed on other cells [38]. This implies they may have specialised functions in bridging innate and adaptive immunity. However, they lack specific markers for monocytes (CD14 and CD16), cDCs (FTL3 and IRF8), moDCs, (CD1A and ITGAM), macrophages (MRC1 and CXCL10), or myeloid-derived suppressor cells (FUT3 and NCAM). Thus, these cell clusters may represent a novel cell type present in cattle and, considering their apparent involvement in vaccine responses, are of interest for further investigation and characterisation.

Differences between bovine and human PBMCs were also observed in other cell populations. For instance, a significant proportion of bovine B cells express SOX5, a gene involved in terminal B cell differentiation [31], an observation also seen in chicken peripheral blood leukocytes [3]. We also identified several NKT cell clusters which, based on their gene expression, suggests that different NKT subsets play diverse roles in the peripheral immune system. For instance, clusters NKT1 and NKT2 expressed high SELL, an adhesion molecule involved in cell trafficking and previously reported on NKT cells with high proliferative potential [39]. Subtypes NKT0, NKT3, and NKT4 also expressed THEMIS, which is involved the effector functions of T cells [40]. This may indicate that THEMIS-positive NKT cells likely retain a stronger dependency on TCR-mediated antigen recognition and may play more adaptive-like roles in immune regulation and response, whereas THEMIS-negative subtypes NKT1 and NKT2, act more like innate effectors. Interestingly, NKT0, NKT1, and NKT2 uniquely express FCER1G, an Fc receptor gamma chain involved in immune cell activation, suggesting that they rely on Fc receptor signalling pathways to initiate immune responses, more akin to NK cells. The GNLY (granulysin) gene, a cytolytic effector protein, was expressed in NKT0 and NKT3, suggesting these subtypes may play roles in direct cytotoxicity. Additionally, NKT3 also uniquely expressed ZBTB16, a transcription factor linked to NKT cell development and survival, along with high levels of IL7R, implicating a role in long-term survival and responsiveness to IL-7 signalling in this subtype.

We identified differences in cell population abundance and transcriptional activity in γδ T cell populations between animals with divergent DTH responses. γδ T cells differ from traditional conventional T cells in that they express semi-invariant γδ T cell receptor (TCR) chains, in contrast to the more variable αβ TCR of conventional T cells [41]. Antigen recognition by the γδ TCR is MHC-independent, allowing γδ T cells to recognise non-peptidyl molecules, such as lipids and other danger or stress-related molecules [41]. The unique features of γδ T cells enable them to perform both innate-like and adaptive-like functions, including specific subsets functioning as professional antigen presenting cells. Indeed, γδ T cells have been highlighted previously as likely key players in vaccination responses in diverse animals. It has been observed that Holstein cattle with a high Cell-IR/low Ab-IR phenotype have higher baseline WC1+ γδ T cell proportions to cattle with a high Ab-IR/low Cell-IR phenotype [42]. Expansion of CD8+ γδ T cells have been documented in chickens following vaccination for *Salmonella Typhimurium* and Marek’s disease virus [43–45]. Roles for γδ T cells in multiple livestock species (cows, sheep, pigs, and goats) have also been proposed in infection and vaccination response [46]. γδ T cells may also possess memory-like functions, as γδ T cells obtained from *Leptospira* vaccinated cattle showed increased responsiveness when exposed to antigen *in vitro* compared to cells from naïve cattle [47].

We have further characterised the subsets of γδ T cells involved in responses to vaccination, whereby elevated levels of CD8- γδ T cells were characteristic of animals with higher Cell-IR phenotypes. Transcriptional analysis of CD8+ γδ T cells suggests that they possess a significantly different phenotype to CD8- γδ T in cattle. Helios, a marker associated with thymic derived T regulatory (Treg) cells [48], was found to be exclusively expressed in CD8+ γδ T cells. This, along with MK expression, which is associated with reduced cytotoxicity [49], supports the potential immunosuppressive role of CD8+ γδ T cells in the peripheral immune system, despite the expression of CD8. Thus, although γδ T cell appear to play an integral role in responses to vaccination generally, specific subsets appear to contribute to the magnitude of responses, highlighting the importance of investigating immune cell subsets in vaccine evaluation, rather than a generalised categorisation of cell types.

In addition to elevated numbers of CD8- γδ T cells in high versus low Cell-IR phenotype animals, these cells exhibited different gene expression profiles. Pathway analysis revealed several pathways involved in immune cell activation, namely those related to mRNA metabolism, translation, proliferation, and adhesion. This suggests that γδ T cells of high responders, specifically the CD8- subset, more readily respond to immune stimulation compared to low responders. Greater insights lie within the broad pathways of ‘signalling’ and ‘response to stimuli’, with more concise analysis of differentially expressed genes involved in positive regulation of T-helper 1 (Th1) cell differentiation and NOD1 signalling regulation, denoted by genes RIPK2 and NFKBIA. RIPK2 was a downregulated gene common to both these pathways. This kinase interacts with the pattern recognition receptor (PRR) NOD1 to promote activation of NFκB signalling following detection of its ligand, bacterial peptidoglycans, leading to production of proinflammatory cytokines, such as TNFα [50, 51]. In addition to these innate immune functions involved in pathogen detection, NOD1 contributes to adaptive immunity via priming to T cell responses and enhancing antibody production [52]. RIPK2 has also been implicated in transduction of TCR signalling, though not specifically in γδ T cells [53]. Both *Clostridium* and *Leptospira* peptidoglycan is detected by NOD1 in mice [54, 55] and, therefore, this pathway is likely important in responses to these pathogens. The other DE gene attributed to NOD1 signalling was *NFKBIA*, which encodes NFκB inhibitor alpha (IκB), the inhibitory component to the NFκB complex that is degraded in response to NOD1 signalling to induce cytokine expression. Downregulation of both these genes would seem paradoxical as diminished expression of RIPK2 would be expected to inhibit NOD1 signalling, while reduced levels of NFKBIA would likely promote NFκB signalling. NFκB, on the other hand, is involved in a variety of pathways to elicit proinflammatory responses, thus while NOD1 signalling is shutdown, perpetuation of inflammation elicited by other pathways is necessary for a robust response to vaccination.

In relation to Th1 differentiation, the role of RIPK2 is unclear, studies have proposed that T cells from RIPK2-KO mice induce differentiation into a pathogenic Th17 lineage leading to excessive IL-17A production [56, 57] and inhibits Th1 differentiation [58]. Others, however, indicate no impact on T cell Th1/Th2 differentiation [59, 60]. The possibility that RIPK2 has distinct functions in γδ T cells is noteworthy. ANXA1 was found to be upregulated in CD8- γδ T cells. ANXA1 expression is regulated by glucocorticoids, with Ca^2+^ binding induces conformational changes to promote phospholipid binding and receptor association, carrying out its function following secretion from the producing cells and acting in a paracrine or autocrine manner [61]. Functions for ANXA1 in innate immunity involve anti-inflammatory activity through association with formyl peptide receptors (FPRs) on neutrophils and monocytes; additional pro-inflammatory roles of ANXA1 have also been proposed [61]. Roles in adaptive immunity are less clear, though addition of recombinant ANXA1 to naïve T cells modulates TCR activation and promotes a Th1 phenotype, while ANXA1 deficient T cells are skewed towards a Th2 phenotype [62]. This suggests that CD8- γδ T cells may promote Th1 responses following vaccination. Furthermore, genes involved in ‘positive regulation of T-helper 1 (Th1) cell differentiation’ were also identified. As the proportion of CD8- γδ T cells were significantly elevated in high Cell-IR phenotype animals, these combined findings suggest that CD8- γδ T cells are expected to play an important role in promoting enhanced Th1 responses in high Cell-IR phenotype animals.

Three clusters of monocytes were found to be upregulated in high responders, namely cMo, ncMo and ML-like. Although strong phagocytic activity is observed by both human and cattle classical monocytes, those from cattle provide the strongest proinflammatory response of monocyte subsets, consistent with our data which demonstrated high IL-1β and CXCL8 expression by this cluster [63]. When compared to low responders, expression of IL-1β and the IL-1β receptor accessory protein-encoding IL1RAP in high responders was upregulated and, to a lesser extent, TNF receptor component TNFRSF1A. This was supported by the CellChat analysis that identified IL-1β–IL-1R1 ligand-receptor interactions as the predominant signalling pathway, originating from and targeting CD14+ monocytes and ML-like cells. Thus, the degree of inflammation may be critical to appropriate responses to vaccination. Previous studies have shown that bovine ncMo exhibit poorer phagocytic activity and, in contrast to human non-classical monocytes, are insensitive to LPS and exhibit reduced expression of neutrophil attractants [63]. Consistent with human monocytes, we found upregulation of complement components C1QA, C1QB and C1QB [64]. However, the lack of phagocytic activity of bovine ncMo suggest they may work in concert with cMo to perform complement-mediated phagocytosis. Bovine ncMO have also been observed to have elevated expression of MHCII compared to other monocytes [63, 64].

In contrast to γδ T cells and monocytes, larger numbers of NKT cells were observed in low responders. NKT cells are another ‘unconventional’ T cell subset that detects foreign antigens in an MHC-independent manner, identifying lipid antigens presented on the MHC-like molecule CD1d expressed on APCs [65]. Activation of NKT cells primarily results in the release of IFNγ and IL-4, in addition to other Th1 and Th2 cytokines, promoting the recruitment and activation of NK cells, T cells and B cells [66]. Conversely, NKT have also been implicated in immunosuppression, particularly during bacterial sepsis, whereby they may also contribute to pathogenesis through controlling inflammation [66–69]. NKT are thought to be associated with strong vaccine responses, with addition to NKT cell agonists proposed to act as vaccine adjuvants to enhance their ‘helper’ functions in promoting adaptive immunity [70, 71]. Thus, the association of low NKT cell populations with high vaccine response observed in the current study is perplexing. Focusing on DE genes expressed by NKT cells in low responders is suggestive of Th1 polarisation. IFITM3 encodes IFN-stimulated gene (ISG) induced by type-I IFN expression that has functions restricting entry of a variety of viruses [72–76]. IFITM3 does not appear to have functions in bacterial immunity but has been reported to promote infection by the intracellular pathogen *Listeria monocytogenes* [77]; upregulation of this gene, therefore, is potentially a byproduct of strong type-I IFN signalling in low responders. JSP.1 is part of the bovine MHC class I complex, which in humans is upregulated in response to type-I IFNs, further supporting elevated expression of these cytokines in low responders [78, 79]. ANXA1 also promotes Th1 differentiation as discussed above. Conversely, NKT cells from high responders upregulated genes that appear to be involved in immune suppression including VWA2, PAPPA and CPT1C. Although specific roles in immune cells remain to be elucidated, these factors have been found to be upregulated in cancer cells and are associated with immune evasion [80–82].

In relation to secreted factors, macrophage migratory inhibitory factor (MIF) and IL-32 were expressed at higher levels in low responders compared to high responders, both of which are proinflammatory cytokines [83, 84]. MIF is expressed constitutively and stored intracellularly by a variety of immune cells, including monocytes/macrophages, B and T lymphocytes, and non-immune, including endocrine, epithelial and endothelial, cell types, providing a rapid response to inflammatory/stress stimuli such LPS, TNFα, and hypoxia [83, 85–87]. This is in addition to chemokine-like functions in promoting recruitment of immune cells to sites of infection/inflammation [88]. Involvement of MIF in vaccine responses has not been well elucidated, though single-nucleotide polymorphisms (SNPs) in MIF correlated with long term maintenance of antibody titres following COVID-19 vaccination [89]; this finding does not reveal whether the activity of MIF promotes or antagonises vaccine responses. MIF has, however, been shown to be associated with the severity of several inflammatory diseases, including sepsis, meningitis and rheumatoid arthritis [85, 90, 91]. IL-32 is expressed in up to nine different isoforms that have varying proinflammatory potencies and share many characteristics of MIF, including expression in immune and non-immune cell types that are induced by similar inflammatory/stress stimuli [84, 92–94]. IL-32 is similarly associated with chronic inflammatory diseases, including rheumatoid arthritis and chronic obstructive pulmonary disease [95, 96]. Intriguingly, IL-32 isoforms have opposing effects on sepsis, with IL-32γ associated with protection and IL-32β associated with increased severity [97, 98]; potentially associated with contrasting anti-inflammatory functions of IL-32 [84].

While this study provides a comprehensive analysis of PBMC transcriptional landscapes, the small sample size (*n* = 8) and reliance on a single breed limit the generalizability and statistical power of our findings. We also looked at a single timepoint post-vaccination, which means cells will be in varying stages of activation, limiting the ability to compare to unstimulated cells or infected cells. The vaccine used is also a cocktail of different toxoid antigens and contains an adjuvant, so pinpointing the specific component of the vaccine that is triggering the differences in immune response is impossible using the methods employed. Nevertheless, this vaccine has been successfully used to induce measurable immune responses to immune competence phenotype Angus beef cattle and phenotypes have been shown to be predictive of feedlot health and productivity outcomes [12]. To strengthen future mechanistic insights, controlled studies using monovalent formulations or defined antigens would be beneficial. Longitudinal studies tracking transcriptional and functional changes in PBMC populations before and following vaccination could also provide deeper insights into the temporal dynamics of immune responses. Additionally, integrating scRNA-seq data with proteomic and metabolomic approaches may uncover additional layers of immune regulation.

In summary, our data suggests two potential differences in the immune responses between high and low cell-mediated responders: (1) low responders have an elevated inflammatory response in NKT cells and (2) high responders have elevated CD8- γδ T cell and pro-inflammatory myeloid activity. Elevated MIF and IL-32 in low responders could suggest an excessive inflammatory response is detrimental to vaccine responses in cattle. Indeed, chronic or enhanced baseline inflammation are associated with poor vaccine responses, and can trigger immunosenescence, more generally [99–101]. Thus, we could hypothesise that low responders may have had higher baseline inflammation pre-vaccination, and the finding of high NKT cell abundance could suggest immune modulation rather than activation. Our second finding highlights the importance of the innate functions of myeloid and Th1 responses of CD8- γδ T cell in the coordination of a robust DTH response. By building our understanding of the cellular makeup of the bovine immune system, it’s varied responses to stimuli, and the intercellular communication, we are creating a foundation for sustainable livestock production; healthier, more disease resilient cattle will lead to increased meat yield, reduced economic losses, and improved food security.

## Electronic supplementary material

Below is the link to the electronic supplementary material.

Supplementary Material 1

Supplementary Material 2 Fig. S1 Predicted source and target of cytokine and chemokine signalling between bovine PBMCs based on ligand-receptor expression patterns

Supplementary Material 3

## Data Availability

All sequencing data generated in this study were submitted to NCBI’s Sequence Read Archive (SRA) under BioProject PRJNA1236904 (BioSample Accessions SAMN47408786– SAMN47408793).

## References

[1] Kappes A, Tozooneyi T, Shakil G, Railey AF, McIntyre KM, Mayberry DE, et al. Livestock health and disease economics: a scoping review of selected literature. Front Vet Sci. 2023;10:1168649. 37795016 10.3389/fvets.2023.1168649 PMC10546065

[2] Vlasova AN, Saif LJ. Bovine immunology: implications for dairy cattle. Front Immunol. 2021;12:643206. 34267745 10.3389/fimmu.2021.643206 PMC8276037

[3] Maxwell M, Söderlund R, Härtle S, Wattrang E. Single-cell RNA-seq mapping of chicken peripheral blood leukocytes. BMC Genomics. 2024;25(1):124. 38287279 10.1186/s12864-024-10044-4 PMC10826067

[4] Wiarda JE, Trachsel JM, Sivasankaran SK, Tuggle CK, Loving CL. Intestinal single-cell atlas reveals novel lymphocytes in pigs with similarities to human cells. Life Sci Alliance. 2022;5(10):e202201442 10.26508/lsa.202201442 PMC9396248 35995567

[5] Wiarda JE, Davila KMS, Trachsel JM, Loving CL, Boggiatto P, Lippolis JD, et al. Single-cell RNA sequencing characterization of Holstein cattle blood and milk immune cells during a chronic *Staphylococcus aureus* mastitis infection. Sci Rep. 2025;15(1):12689. 40221598 10.1038/s41598-025-96657-5 PMC11993596

[6] Gao Y, Li J, Cai G, Wang Y, Yang W, Li Y, et al. Single-cell transcriptomic and chromatin accessibility analyses of dairy cattle peripheral blood mononuclear cells and their responses to lipopolysaccharide. BMC Genomics. 2022;23(1):338. 35501711 10.1186/s12864-022-08562-0 PMC9063233

[7] Barut GT, Kreuzer M, Bruggmann R, Summerfield A, Talker SC. Single-cell transcriptomics reveals striking heterogeneity and functional organization of dendritic and monocytic cells in the bovine mesenteric lymph node. Front Immunol. 2022;13:1099357. 36685557 10.3389/fimmu.2022.1099357 PMC9853064

[8] Zorc M, Dolinar M, Dovč P. A single-cell transcriptome of bovine milk somatic cells. Genes (Basel). 2024;15(3):349 10.3390/genes15030349 PMC10970057 38540408

[9] Derbois C, Palomares M-A, Deleuze J-F, Cabannes E, Bonnet E. Single cell transcriptome sequencing of stimulated and frozen human peripheral blood mononuclear cells. Sci Data. 2023;10(1):433. 37414801 10.1038/s41597-023-02348-z PMC10326076

[10] Guzman E, Hope J, Taylor G, Smith AL, Cubillos-Zapata C, Charleston B. Bovine γδ T cells are a major regulatory T cell subset. J Immunol. 2014;193(1):208–22. 24890724 10.4049/jimmunol.1303398 PMC4065783

[11] Husseini N, Beard SC, Hodgins DC, Barnes C, Chik E, Mallard BA. Immuno-phenotyping of Canadian beef cattle: adaptation of the high immune response methodology for utilization in beef cattle. Transl Anim Sci. 2022;6(1):txac006. 10.1093/tas/txac006 PMC8896012 35261968

[12] Hine BC, Bell AM, Niemeyer DDO, Duff CJ, Butcher NM, Dominik S et al. Associations between immune competence phenotype and feedlot health and productivity in Angus cattle. J Ani Sci. 2021;99(2):skab016. 10.1093/jas/skab016 PMC7901007 33476384

[13] Hine BC, Bell AM, Niemeyer DDO, Duff CJ, Butcher NM, Dominik S, et al. Immune competence traits assessed during the stress of weaning are heritable and favorably genetically correlated with temperament traits in Angus cattle. J Ani Sci. 2019;97(10):4053–65. 10.1093/jas/skz260 PMC6776280 31581299

[14] Reverter A, Hine BC, Porto-Neto L, Li Y, Duff CJ, Dominik S et al. ImmuneDEX: a strategy for the genetic improvement of immune competence in Australian Angus cattle. J Ani Sci. 2021;99(3):skaa384. 10.1093/jas/skaa384 PMC7936916 33677583

[15] Palgen JL, Tchitchek N, Elhmouzi-Younes J, Delandre S, Namet I, Rosenbaum P, et al. Prime and boost vaccination elicit a distinct innate myeloid cell immune response. Sci Rep. 2018;8(1):3087. 29449630 10.1038/s41598-018-21222-2 PMC5814452

[16] Lee G-J, Quan F-S. Protection induced by early stage vaccination with pandemic influenza virus-like particles. Vaccine. 2016;34(33):3764–72. 27317263 10.1016/j.vaccine.2016.06.011

[17] Barrios Y, Franco A, Sánchez-Machín I, Poza-Guedes P, González-Pérez R, Matheu V. The beauty of simplicity: Delayed-Type hypersensitivity reaction to measure cellular immune responses in RNA-SARS-Cov-2 vaccinated individuals. Vaccines. 2021;9(6):575. 34205888 10.3390/vaccines9060575 PMC8227045

[18] Stubbington MJT, Rozenblatt-Rosen O, Regev A, Teichmann SA. Single-cell transcriptomics to explore the immune system in health and disease. Science. 2017;358(6359):58–63. 28983043 10.1126/science.aan6828 PMC5654495

[19] Hao Y, Stuart T, Kowalski MH, Choudhary S, Hoffman P, Hartman A, et al. Dictionary learning for integrative, multimodal and scalable single-cell analysis. Nat Biotechnol. 2024;42(2):293–304. 37231261 10.1038/s41587-023-01767-y PMC10928517

[20] Jin S, Guerrero-Juarez CF, Zhang L, Chang I, Ramos R, Kuan C-H, et al. Inference and analysis of cell-cell communication using cellchat. Nat Commun. 2021;12(1):1088. 33597522 10.1038/s41467-021-21246-9 PMC7889871

[21] Wickham H. ggplot2: elegant graphics for data analysis. New York: Springer-; 2016.

[22] Love MI, Huber W, Anders S. Moderated Estimation of fold change and dispersion for RNA-seq data with DESeq2. Genome Biol. 2014;15(12):550. 25516281 10.1186/s13059-014-0550-8 PMC4302049

[23] Elizarraras JM, Liao Y, Shi Z, Zhu Q, Pico Alexander R, Zhang B. WebGestalt 2024: faster gene set analysis and new support for metabolomics and multi-omics. Nucleic Acids Res. 2024;52(W1):W415–21. 38808672 10.1093/nar/gkae456 PMC11223849

[24] Vanhooren J, Derpoorter C, Depreter B, Deneweth L, Philippé J, De Moerloose B, et al. TARP as antigen in cancer immunotherapy. Cancer Immunol Immun. 2021;70(11):3061–8. 10.1007/s00262-021-02972-x PMC8164403 34050774

[25] Zhao P, Zou J, Zhou F, Zhu Y, Song Q, Yu D, et al. Immune features of COVID-19 convalescent individuals revealed by a single-cell RNA sequencing. Int Immunopharmacol. 2022;108:108767. 35453072 10.1016/j.intimp.2022.108767 PMC9013654

[26] Guerra-Maupome M, Palmer MV, Waters WR, McGill JL. Characterization of γδ T cell effector/memory subsets based on CD27 and CD45R expression in response to *Mycobacterium bovis* infection. Immunohorizons. 2019;3(6):208–18. 31356167 10.4049/immunohorizons.1900032 PMC6875775

[27] Talker SC, Baumann A, Barut GT, Keller I, Bruggmann R, Summerfield A. Precise delineation and transcriptional characterization of bovine blood dendritic-cell and monocyte subsets. Front Immunol. 2018;9:2505. 30425716 10.3389/fimmu.2018.02505 PMC6218925

[28] Shao W, Wang Y, Fang Q, Shi W, Qi H. Epigenetic recording of stimulation history reveals BLIMP1–BACH2 balance in determining memory B cell fate upon recall challenge. Nat Immunol. 2024;25(8):1432–44. 38969872 10.1038/s41590-024-01900-2

[29] Thorarinsdottir K, Camponeschi A, Cavallini N, Grimsholm O, Jacobsson L, Gjertsson I, et al. CD21(-/low) B cells in human blood are memory cells. Clin Exp Immunol. 2016;185(2):252–62. 27010233 10.1111/cei.12795 PMC4955005

[30] Li J, Kurasawa Y, Wang Y, Clise-Dwyer K, Klumpp SA, Liang H, et al. Requirement for ssbp2 in hematopoietic stem cell maintenance and stress response. J Immunol. 2014;193(9):4654–62. 25238756 10.4049/jimmunol.1300337 PMC4201964

[31] Rakhmanov M, Sic H, Kienzler AK, Fischer B, Rizzi M, Seidl M, et al. High levels of SOX5 decrease proliferative capacity of human B cells, but permit plasmablast differentiation. PLoS ONE. 2014;9(6):e100328. 24945754 10.1371/journal.pone.0100328 PMC4063782

[32] Trezise S, Karnowski A, Fedele PL, Mithraprabhu S, Liao Y, D’Costa K et al. Mining the plasma cell transcriptome for novel cell surface proteins. Int J Mol Sci. 2018;19(8):2161. 10.3390/ijms19082161 PMC6121261 30042348

[33] Endsley JJ, Endsley MA, Estes DM. Bovine natural killer cells acquire cytotoxic/effector activity following activation with IL-12/15 and reduce Mycobacterium bovis BCG in infected macrophages. J Leukoc Biol. 2006;79(1):71–9. 16275895 10.1189/jlb.0505239

[34] Kovalovsky D, Uche OU, Eladad S, Hobbs RM, Yi W, Alonzo E, et al. The BTB-zinc finger transcriptional regulator PLZF controls the development of invariant natural killer T cell effector functions. Nat Immunol. 2008;9(9):1055–64. 18660811 10.1038/ni.1641 PMC2662733

[35] Neumaier EE, Rothhammer V, Linnerbauer M. The role of midkine in health and disease. Front Immunol. 2023;14:1310094. 38098484 10.3389/fimmu.2023.1310094 PMC10720637

[36] Fleischer J, Soeth E, Reiling N, Grage-Griebenow E, Flad HD, Ernst M. Differential expression and function of CD80 (B7-1) and CD86 (B7-2) on human peripheral blood monocytes. Immunology. 1996;89(4):592–8. 9014827 10.1046/j.1365-2567.1996.d01-785.x PMC1456589

[37] Riaz B, Islam SMS, Ryu HM, Sohn S. CD83 regulates the immune responses in inflammatory disorders. Int J Mol Sci. 2023;24(3):2831. 10.3390/ijms24032831 PMC9917562 36769151

[38] Barclay AN, Van den Berg TK. The interaction between signal regulatory protein alpha (SIRPα) and CD47: structure, function, and therapeutic target. Annu Rev Immunol. 2014;32:25–50. 24215318 10.1146/annurev-immunol-032713-120142

[39] Tian G, Courtney AN, Jena B, Heczey A, Liu D, Marinova E, et al. CD62L + NKT cells have prolonged persistence and antitumor activity in vivo. J Clin Invest. 2016;126(6):2341–55. 27183388 10.1172/JCI83476 PMC4887157

[40] Prasad M, Brzostek J, Gautam N, Balyan R, Rybakin V, Gascoigne NRJ. Themis regulates metabolic signaling and effector functions in CD4 + T cells by controlling NFAT nuclear translocation. Cell Mol Immuno. 2021;18(9):2249–61. 10.1038/s41423-020-00578-4 PMC8429700 33177694

[41] Hu Y, Hu Q, Li Y, Lu L, Xiang Z, Yin Z, et al. γδ T cells: origin and fate, subsets, diseases and immunotherapy. Signal Transduct Target Ther. 2023;8(1):434. 37989744 10.1038/s41392-023-01653-8 PMC10663641

[42] Hine BC, Cartwright SL, Mallard BA. Analysis of leukocyte populations in Canadian Holsteins classified as high or low immune responders for antibody- or cell-mediated immune response. Can J Vet Res. 2012;76(2):149–56. 23024458 PMC3314438

[43] Berndt A, Methner U. Gamma/delta T cell response of chickens after oral administration of attenuated and non-attenuated *Salmonella typhimurium* strains. Vet Immunol Immunopathol. 2001;78(2):143–61. 11182154 10.1016/s0165-2427(00)00264-6

[44] Matsuyama-Kato A, Iseki H, Boodhoo N, Bavananthasivam J, Alqazlan N, Abdul-Careem MF, et al. Phenotypic characterization of gamma delta (γδ) T cells in chickens infected with or vaccinated against marek’s disease virus. Virology. 2022;568:115–25. 35152043 10.1016/j.virol.2022.01.012

[45] Hao X, Li S, Li J, Yang Y, Qin A, Shang S. An anti-tumor vaccine against marek’s disease virus induces differential activation and memory response of γδ T cells and CD8 T cells in chickens. Front Immunol. 2021;12:645426. 33659011 10.3389/fimmu.2021.645426 PMC7917234

[46] Baldwin CL, Yirsaw A, Gillespie A, Le Page L, Zhang F, Damani-Yokota P, et al. γδ T cells in livestock: responses to pathogens and vaccine potential. Transbound Emerg Dis. 2020;67(Suppl 2):119–28. 31515956 10.1111/tbed.13328

[47] Blumerman SL, Herzig CT, Baldwin CL. WC1 + gammadelta T cell memory population is induced by killed bacterial vaccine. Eur J Immunol. 2007;37(5):1204–16. 17429840 10.1002/eji.200636216

[48] Thornton AM, Lu J, Korty PE, Kim YC, Martens C, Sun PD, et al. Helios(+) and Helios(-) Treg subpopulations are phenotypically and functionally distinct and express dissimilar TCR repertoires. Eur J Immunol. 2019;49(3):398–412. 30620397 10.1002/eji.201847935 PMC6402968

[49] Strijker JGM, Pascual-Pasto G, Grothusen GP, Kalmeijer YJ, Kalaitsidou E, Zhao C, et al. Blocking MIF secretion enhances CAR T-cell efficacy against neuroblastoma. Eur J Cancer. 2025;218:115263. 39908652 10.1016/j.ejca.2025.115263 PMC11884407

[50] Park JH, Kim YG, McDonald C, Kanneganti TD, Hasegawa M, Body-Malapel M, et al. RICK/RIP2 mediates innate immune responses induced through Nod1 and Nod2 but not TLRs. J Immunol. 2007;178(4):2380–6. 17277144 10.4049/jimmunol.178.4.2380

[51] Inohara N, Ogura Y, Chen FF, Muto A, Nuñez G. Human Nod1 confers responsiveness to bacterial lipopolysaccharides. J Biol Chem. 2001;276(4):2551–4. 11058605 10.1074/jbc.M009728200

[52] Fritz JH, Le Bourhis L, Sellge G, Magalhaes JG, Fsihi H, Kufer TA, et al. Nod1-mediated innate immune recognition of peptidoglycan contributes to the onset of adaptive immunity. Immunity. 2007;26(4):445–59. 17433730 10.1016/j.immuni.2007.03.009

[53] Ruefli-Brasse AA, Lee WP, Hurst S, Dixit VM. Rip2 participates in Bcl10 signaling and T-cell receptor-mediated NF-kappaB activation. J Biol Chem. 2004;279(2):1570–4. 14638696 10.1074/jbc.C300460200

[54] Hasegawa M, Yamazaki T, Kamada N, Tawaratsumida K, Kim YG, Núñez G, et al. Nucleotide-binding oligomerization domain 1 mediates recognition of *Clostridium difficile* and induces neutrophil recruitment and protection against the pathogen. J Immunol. 2011;186(8):4872–80. 21411735 10.4049/jimmunol.1003761

[55] Ratet G, Santecchia I, Fanton d’Andon M, Vernel-Pauillac F, Wheeler R, Lenormand P, et al. LipL21 lipoprotein binding to peptidoglycan enables *Leptospira interrogans* to escape NOD1 and NOD2 recognition. PLoS Pathog. 2017;13(12):e1006725. 29211798 10.1371/journal.ppat.1006725 PMC5764436

[56] Shimada K, Porritt RA, Markman JL, O’Rourke JG, Wakita D, Noval Rivas M, et al. T-Cell-Intrinsic receptor interacting protein 2 regulates pathogenic T helper 17 cell differentiation. Immunity. 2018;49(5):873–e885877. 30366765 10.1016/j.immuni.2018.08.022 PMC6260980

[57] Song ZC, Liu ST, Xia XY, Hu JJ, Leng RX, Zhao W. In vitro Silencing of RIP2 in Naive CD4(+) T cells from lupus-prone mice promotes pathogenic Th17 cell differentiation. Clin Rheumatol. 2024;43(11):3515–23. 39235498 10.1007/s10067-024-07124-x

[58] Kobayashi K, Inohara N, Hernandez LD, Galán JE, Núñez G, Janeway CA, et al. RICK/Rip2/CARDIAK mediates signalling for receptors of the innate and adaptive immune systems. Nature. 2002;416(6877):194–9. 11894098 10.1038/416194a

[59] Hall HT, Wilhelm MT, Saibil SD, Mak TW, Flavell RA, Ohashi PS. RIP2 contributes to Nod signaling but is not essential for T cell proliferation, T helper differentiation or TLR responses. Eur J Immunol. 2008;38(1):64–72. 18085666 10.1002/eji.200737393

[60] Fairhead T, Lian D, McCully ML, Garcia B, Zhong R, Madrenas J. RIP2 is required for NOD signaling but not for Th1 cell differentiation and cellular allograft rejection. Am J Transpl. 2008;8(6):1143–50. 10.1111/j.1600-6143.2008.02236.x 18522545

[61] Kelly L, McGrath S, Rodgers L, McCall K, Tulunay Virlan A, Dempsey F, et al. Annexin-A1: the culprit or the solution? Immunology. 2022;166(1):2–16. 35146757 10.1111/imm.13455 PMC9426623

[62] D’Acquisto F, Paschalidis N, Sampaio AL, Merghani A, Flower RJ, Perretti M. Impaired T cell activation and increased Th2 lineage commitment in Annexin-1-deficient T cells. Eur J Immunol. 2007;37(11):3131–42. 17948261 10.1002/eji.200636792

[63] Hussen J, Düvel A, Sandra O, Smith D, Sheldon IM, Zieger P, et al. Phenotypic and functional heterogeneity of bovine blood monocytes. PLoS ONE. 2013;8(8):e71502. 23967219 10.1371/journal.pone.0071502 PMC3743816

[64] Rigamonti A, Castagna A, Viatore M, Colombo FS, Terzoli S, Peano C, et al. Distinct responses of newly identified monocyte subsets to advanced Gastrointestinal cancer and COVID-19. Front Immunol. 2022;13:967737. 36263038 10.3389/fimmu.2022.967737 PMC9576306

[65] Vogt S, Mattner J. NKT cells contribute to the control of microbial infections. Front Cell Infect Microbiol. 2021;11:718350. 34595131 10.3389/fcimb.2021.718350 PMC8477047

[66] Liao CM, Zimmer MI, Wang CR. The functions of type I and type II natural killer T cells in inflammatory bowel diseases. Inflamm Bowel Dis. 2013;19(6):1330–8. 23518808 10.1097/MIB.0b013e318280b1e3 PMC3694171

[67] Rhee RJ, Carlton S, Lomas JL, Lane C, Brossay L, Cioffi WG, et al. Inhibition of CD1d activation suppresses septic mortality: a role for NK-T cells in septic immune dysfunction. J Surg Res. 2003;115(1):74–81. 14572776 10.1016/s0022-4804(03)00220-8

[68] Anantha RV, Mazzuca DM, Xu SX, Porcelli SA, Fraser DD, Martin CM, et al. T helper type 2-polarized invariant natural killer T cells reduce disease severity in acute intra-abdominal sepsis. Clin Exp Immunol. 2014;178(2):292–309. 24965554 10.1111/cei.12404 PMC4233379

[69] Hu CK, Venet F, Heffernan DS, Wang YL, Horner B, Huang X, et al. The role of hepatic invariant NKT cells in systemic/local inflammation and mortality during polymicrobial septic shock. J Immunol. 2009;182(4):2467–75. 19201902 10.4049/jimmunol.0801463 PMC2653268

[70] Burn OK, Pankhurst TE, Painter GF, Connor LM, Hermans IF. Harnessing NKT cells for vaccination. Oxf Open Immunol. 2021;2(1):iqab013. 36845569 10.1093/oxfimm/iqab013 PMC9914585

[71] Mattarollo SR, Smyth MJ. NKT cell adjuvants in therapeutic vaccines against hematological cancers. Oncoimmunology. 2013;2(1):e22615. 23482240 10.4161/onci.22615 PMC3583928

[72] Spence JS, He R, Hoffmann HH, Das T, Thinon E, Rice CM, et al. IFITM3 directly engages and shuttles incoming virus particles to lysosomes. Nat Chem Biol. 2019;15(3):259–68. 30643282 10.1038/s41589-018-0213-2 PMC6466627

[73] Klein S, Golani G, Lolicato F, Lahr C, Beyer D, Herrmann A, et al. IFITM3 blocks influenza virus entry by sorting lipids and stabilizing hemifusion. Cell Host Microbe. 2023;31(4):616–e633620. 37003257 10.1016/j.chom.2023.03.005

[74] Du S, Wang Y, Wang J, Ma Y, Xu W, Shi X, et al. IFITM3 inhibits severe fever with thrombocytopenia syndrome virus entry and interacts with viral Gc protein. J Med Virol. 2024;96(3):e29491. 38402626 10.1002/jmv.29491

[75] Franz S, Pott F, Zillinger T, Schüler C, Dapa S, Fischer C et al. Human IFITM3 restricts Chikungunya virus and Mayaro virus infection and is susceptible to virus-mediated counteraction. Life Sci Alliance. 2021;4(7):e202000909. 10.26508/lsa.202000909 PMC8200292 34078739

[76] McMichael TM, Zhang Y, Kenney AD, Zhang L, Zani A, Lu M, et al. IFITM3 restricts human metapneumovirus infection. J Infect Dis. 2018;218(10):1582–91. 29917090 10.1093/infdis/jiy361 PMC6173576

[77] Tan JMJ, Garner ME, Regeimbal JM, Greene CJ, Márquez JDR, Ammendolia DA, et al. Listeria exploits IFITM3 to suppress antibacterial activity in phagocytes. Nat Commun. 2021;12(1):4999. 34404769 10.1038/s41467-021-24982-0 PMC8371165

[78] Ljunggren G, Anderson DJ. Cytokine induced modulation of MHC class I and class II molecules on human cervical epithelial cells. J Reprod Immunol. 1998;38(2):123–38. 9730287 10.1016/s0165-0378(98)00009-6

[79] Coomans de Brachène A, Dos Santos RS, Marroqui L, Colli ML, Marselli L, Mirmira RG, et al. IFN-α induces a Preferential long-lasting expression of MHC class I in human pancreatic beta cells. Diabetologia. 2018;61(3):636–40. 29305625 10.1007/s00125-017-4536-4 PMC6241216

[80] Tian Y, Zhao Q, Wu H, Guo J, Wu H. VWA2 protein molecular mechanism predicts colorectal cancer: promoting cell invasion and migration by inhibiting NK cell activation. Int J Biol Macromol. 2024;279(Pt 3):135394. 39245093 10.1016/j.ijbiomac.2024.135394

[81] Heitzeneder S, Sotillo E, Shern JF, Sindiri S, Xu P, Jones R, et al. Pregnancy-associated plasma Protein-A (PAPP-A) in ewing sarcoma: role in tumor growth and immune evasion. J Natl Cancer Inst. 2019;111(9):970–82. 30698726 10.1093/jnci/djy209 PMC6748813

[82] Wei R, Song J, Pan H, Liu X, Gao J. CPT1C-positive cancer-associated fibroblast facilitates immunosuppression through promoting IL-6-induced M2-like phenotype of macrophage. Oncoimmunology. 2024;13(1):2352179. 38746869 10.1080/2162402X.2024.2352179 PMC11093039

[83] Grieb G, Merk M, Bernhagen J, Bucala R. Macrophage migration inhibitory factor (MIF): a promising biomarker. Drug News Perspect. 2010;23(4):257–64. 20520854 10.1358/dnp.2010.23.4.1453629 PMC3131110

[84] Aass KR, Kastnes MH, Standal T. Molecular interactions and functions of IL-32. J Leukoc Biol. 2021;109(1):143–59. 32869391 10.1002/JLB.3MR0620-550R

[85] Calandra T, Bernhagen J, Metz CN, Spiegel LA, Bacher M, Donnelly T, et al. MIF as a glucocorticoid-induced modulator of cytokine production. Nature. 1995;377(6544):68–71. 7659164 10.1038/377068a0

[86] Simons D, Grieb G, Hristov M, Pallua N, Weber C, Bernhagen J, et al. Hypoxia-induced endothelial secretion of macrophage migration inhibitory factor and role in endothelial progenitor cell recruitment. J Cell Mol Med. 2011;15(3):668–78. 20178462 10.1111/j.1582-4934.2010.01041.x PMC3922388

[87] Lan HY, Yang N, Metz C, Mu W, Song Q, Nikolic-Paterson DJ, et al. TNF-alpha up-regulates renal MIF expression in rat crescentic glomerulonephritis. Mol Med. 1997;3(2):136–44. 9085256 PMC2230061

[88] Bernhagen J, Krohn R, Lue H, Gregory JL, Zernecke A, Koenen RR, et al. MIF is a noncognate ligand of CXC chemokine receptors in inflammatory and atherogenic cell recruitment. Nat Med. 2007;13(5):587–96. 17435771 10.1038/nm1567

[89] Takemoto Y, Tanimine N, Yoshinaka H, Tanaka Y, Takafuta T, Sugiyama A, et al. Multi-phasic gene profiling using candidate gene approach predict the capacity of specific antibody production and maintenance following COVID-19 vaccination in Japanese population. Front Immunol. 2023;14:1217206. 37564647 10.3389/fimmu.2023.1217206 PMC10411726

[90] Østergaard C, Benfield T. Macrophage migration inhibitory factor in cerebrospinal fluid from patients with central nervous system infection. Crit Care. 2009;13(3):R101. 19558639 10.1186/cc7933 PMC2717473

[91] Leech M, Metz C, Hall P, Hutchinson P, Gianis K, Smith M, et al. Macrophage migration inhibitory factor in rheumatoid arthritis: evidence of Proinflammatory function and regulation by glucocorticoids. Arthritis Rheum. 1999;42(8):1601–8. 10446857 10.1002/1529-0131(199908)42:8<1601::AID-ANR6>3.0.CO;2-B

[92] Shoda H, Fujio K, Yamaguchi Y, Okamoto A, Sawada T, Kochi Y, et al. Interactions between IL-32 and tumor necrosis factor alpha contribute to the exacerbation of immune-inflammatory diseases. Arthritis Res Ther. 2006;8(6):R166. 17078892 10.1186/ar2074 PMC1794509

[93] Nakayama M, Niki Y, Kawasaki T, Takeda Y, Ikegami H, Toyama Y, et al. IL-32-PAR2 axis is an innate immunity sensor providing alternative signaling for LPS-TRIF axis. Sci Rep. 2013;3:2960. 24129891 10.1038/srep02960 PMC3797434

[94] Zahoor M, Westhrin M, Aass KR, Moen SH, Misund K, Psonka-Antonczyk KM, et al. Hypoxia promotes IL-32 expression in myeloma cells, and high expression is associated with poor survival and bone loss. Blood Adv. 2017;1(27):2656–66. 29296919 10.1182/bloodadvances.2017010801 PMC5745138

[95] Alsaleh G, Sparsa L, Chatelus E, Ehlinger M, Gottenberg JE, Wachsmann D, et al. Innate immunity triggers IL-32 expression by fibroblast-like synoviocytes in rheumatoid arthritis. Arthritis Res Ther. 2010;12(4):R135. 20615213 10.1186/ar3073 PMC2945025

[96] Calabrese F, Baraldo S, Bazzan E, Lunardi F, Rea F, Maestrelli P, et al. IL-32, a novel Proinflammatory cytokine in chronic obstructive pulmonary disease. Am J Respir Crit Care Med. 2008;178(9):894–901. 18703789 10.1164/rccm.200804-646OC

[97] Kim SJ, Lee S, Kwak A, Kim E, Jo S, Bae S, et al. Interleukin-32γ Transgenic mice resist LPS-mediated septic shock. J Microbiol Biotechnol. 2014;24(8):1133–42. 24743568 10.4014/jmb.1404.04012

[98] Kobayashi H, Huang J, Ye F, Shyr Y, Blackwell TS, Lin PC. Interleukin-32beta propagates vascular inflammation and exacerbates sepsis in a mouse model. PLoS ONE. 2010;5(3):e9458. 20221440 10.1371/journal.pone.0009458 PMC2832764

[99] Pereira B, Xu XN, Akbar AN. Targeting inflammation and Immunosenescence to improve vaccine responses in the elderly. Front Immunol. 2020;11:583019. 33178213 10.3389/fimmu.2020.583019 PMC7592394

[100] Alter G, Sekaly RP. Beyond adjuvants: antagonizing inflammation to enhance vaccine immunity. Vaccine. 2015;33(Suppl 2):B55–59. 26022570 10.1016/j.vaccine.2015.03.058

[101] Hou J, Wang S, Li D, Carpp LN, Zhang T, Liu Y, et al. Early pro-inflammatory signal and T-Cell activation associate with vaccine-induced anti-vaccinia protective neutralizing antibodies. Front Immunol. 2021;12:737487. 34707608 10.3389/fimmu.2021.737487 PMC8542877

