# Supplementary material for: Single-cell transcriptomics uncovers key immune drivers of vaccine efficacy in cattle
Source: BMC Genomics. 2025 Aug 18;26:750. doi: 10.1186/s12864-025-11915-0 (PMC12359925; doi:10.1186/s12864-025-11915-0)
Supplement: Supplementary file 1 — Supplementary Material 1 [file 12864_2025_11915_MOESM1_ESM.docx]

Supplementary Table 1: Sequencing data generated using scRNAseq

| Animal | Immune Response | No. cells | Reads per cell | Genes per cell |
| --- | --- | --- | --- | --- |
| 1 | Low | 6,114 | 32,300 | 1,921 |
| 2 | Low | 3,219 | 12,059 | 1,591 |
| 3 | Low | 4,078 | 11,602 | 1,551 |
| 4 | Low | 3,821 | 17,550 | 1,897 |
| 5 | High | 5,173 | 31,807 | 1,845 |
| 6 | High | 2,853 | 19,292 | 1,752 |
| 7 | High | 4,085 | 12,288 | 1,621 |
| 8 | High | 3,104 | 17,363 | 1,901 |
|  |  | 32,447 | 19,282 | 1,760 |

Supplementary Table 2: Differences in the proportion of cells in clusters between high and low Cell-IR animals

| Cluster | Log2FD | P value | FDR-adj P value |
| --- | --- | --- | --- |
| CD8- γδ T | -0.696 | 0.001 | 0.002 |
| myeloid-like | -1.064 | 0.001 | 0.002 |
| CD16+ | -0.967 | 0.001 | 0.002 |
| CD14+ | -0.386 | 0.001 | 0.002 |
| NKT | 1.073 | 0.001 | 0.002 |
| B | 0.442 | 0.001 | 0.002 |
| NK | 0.191 | 0.002 | 0.003 |
| Proliferating cells | 0.463 | 0.001 | 0.002 |
| DN T | 0.534 | 0.025 | 0.035 |
| CD4+ T | 0.127 | 0.002 | 0.003 |
| Plasma | 0.225 | 0.192 | 0.224 |
| DC | -0.120 | 0.430 | 0.430 |
| CD8+ γδ T | -0.043 | 0.283 | 0.304 |
| CD8+ T | -0.156 | 0.087 | 0.111 |
