# Supplementary figures and images for: Single-cell transcriptomics uncovers key immune drivers of vaccine efficacy in cattle
Source: BMC Genomics. 2025 Aug 18;26:750. doi: 10.1186/s12864-025-11915-0 (PMC12359925; doi:10.1186/s12864-025-11915-0)

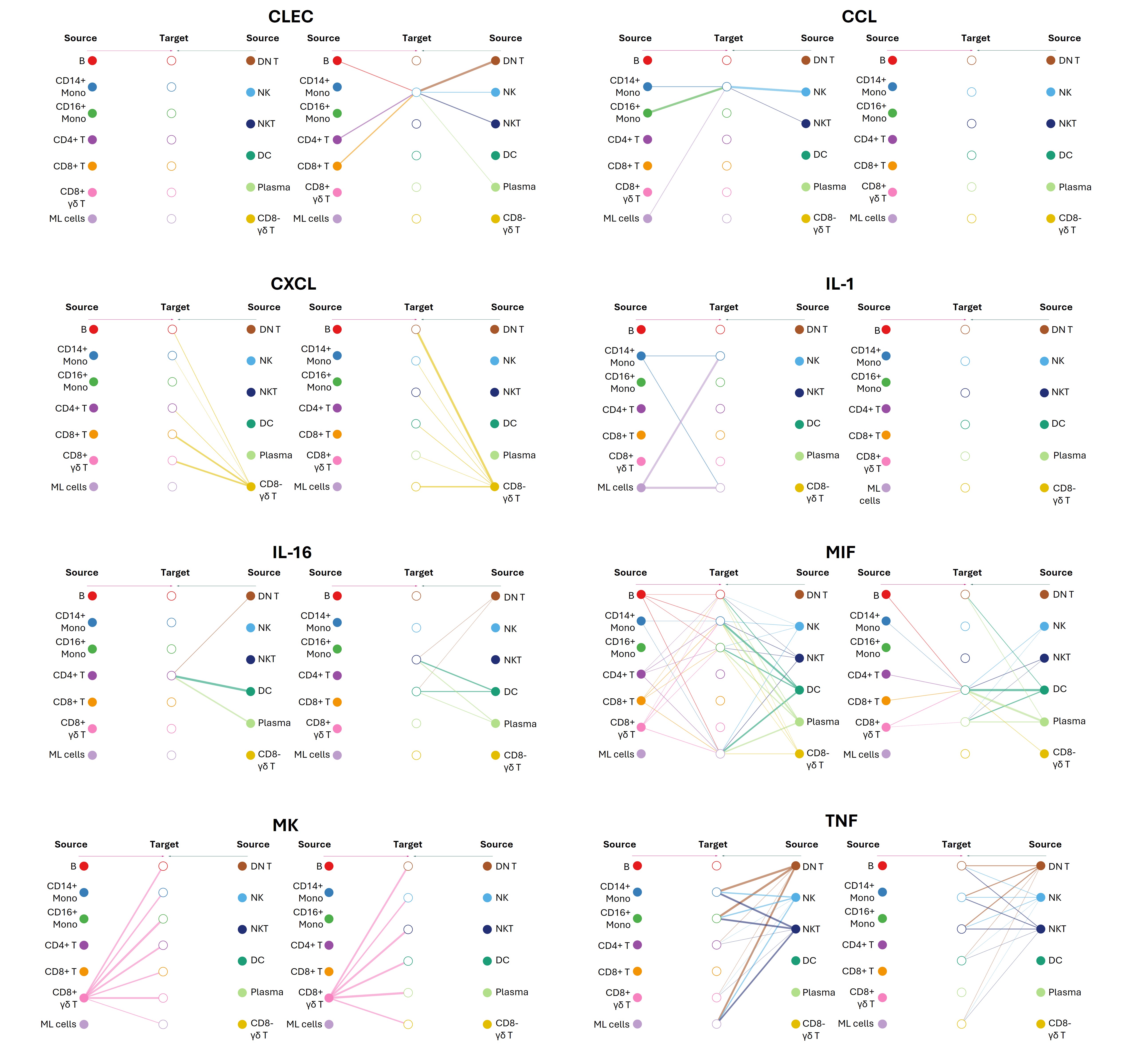

Supplement: Supplementary file 2 — Supplementary Material 2 Fig. S1 Predicted source and target of cytokine and chemokine signalling between bovine PBMCs based on ligand-receptor expression patterns [file 12864_2025_11915_MOESM2_ESM.jpg]
